# Key regulatory pathways, microRNAs, and target genes participate in adventitious root formation of *Acer rubrum* L

**DOI:** 10.1038/s41598-022-16255-7

**Published:** 2022-07-14

**Authors:** Wenpeng Zhu, Manyu Zhang, Jianyi Li, Hewen Zhao, Kezhong Zhang, Wei Ge

**Affiliations:** 1 Beijing Advanced Innovation Center for Tree Breeding By Molecular Design, Beijing University of Agriculture, Beijing, 102206 People’s Republic of China; 2 College of Landscape Architecture, Beijing University of Agriculture, Beijing, 102206 People’s Republic of China; 3 Beijing Laboratory of Urban and Rural Ecological Environment, Beijing, 102206 People’s Republic of China

**Keywords:** Developmental biology, Molecular biology, Plant sciences

## Abstract

Red maple (*Acer rubrum* L.) is a type of colorful ornamental tree with great economic value. Because this tree is difficult to root under natural conditions and the seedling survival rate is low, vegetative propagation methods are often used. Because the formation of adventitious roots (ARs) is essential for the asexual propagation of *A. rubrum*, it is necessary to investigate the molecular regulatory mechanisms of AR formation in *A. rubrum*. To address this knowledge gap, we sequenced the transcriptome and small RNAs (sRNAs) of the *A. rubrum* variety ‘Autumn Fantasy’ using high-throughput sequencing and explored changes in gene and microRNA (miRNA) expression in response to exogenous auxin treatment. We identified 82,468 differentially expressed genes (DEGs) between the treated and untreated ARs, as well as 48 known and 95 novel miRNAs. We also identified 172 target genes of the known miRNAs using degradome sequencing. Two key regulatory pathways (ubiquitin mediated proteolysis and plant hormone signal transduction), *Ar-miR160a* and the target gene *auxin response factor 10* (*ArARF10*) were selected based on KEGG pathway and cluster analyses. We further investigated the expression patterns and regulatory roles of *ArARF10* through subcellular localization, transcriptional activation, plant transformation, qRT-PCR analysis, and GUS staining. Experiments overexpressing *ArARF10* and *Ar-miR160a*, indicated that *ArARF10* promoted AR formation, while *Ar-miR160a* inhibited AR formation. Transcription factors (TFs) and miRNAs related to auxin regulation that promote AR formation in *A. rubrum* were identified. Differential expression patterns indicated the *Ar-miR160a*-*ArARF10* interaction might play a significant role in the regulation of AR formation in *A. rubrum*. Our study provided new insights into mechanisms underlying the regulation of AR formation in *A. rubrum*.

## Introduction

*Acer rubrum*, a large deciduous tree in family Aceraceae Juss^1^, is often used to beautify urban gardens due to its strong adaptability and rapid growth^2^. However, this tree struggle to root under natural conditions, and the survival rate of naturally rooted seedlings is low^3^. Therefore, some red maple varieties, such as the *A. rubrum* hybrid ‘Autumn Fantasy’, are primarily vegetatively propagated using softwood cuttings^1,4^.

AR formation is a critical aspect of cultivation for most forest species that are vegetatively propagated from elite genotypes^5^. AR formation is a developmental process wherein new roots are generated spontaneously (or in response to certain stimuli) from stems, leaves, or the non-pericycle tissues of older roots^6^. In particular, AR formation provides the basis for clonal multiplication, a technique that is utilized for the breeding and propagation of many crop and forestry plants^7^. Auxin, which is formed in roots and stems, and which is involved in stem cell maintenance and differentiation, effectively induces AR formation^8,9^. Auxin dosage, gradient, and response are all important for plant root growth^10^. Synthetic auxins, such as indole-3-butyric acid (IBA), have been used to induce AR for almost 80 years^11,12^. However, the molecular mechanisms regulating hormone-induced AR development in forest tree species remain incompletely understood.

Although molecular studies have identified several genes associated with AR in model plant species, such as *Arabidopsis thaliana* and *Populus*^6,13^, only a few genes that regulate AR formation have been identified in horticultural plants^14^, and most of these encode TFs^14^. In total, 35 genes encoding TFs were shown to exhibit significant changes in expression level during AR growth and development in *Populus*^15^. However, the functions of most of these genes remain unknown.

By contrast, several genes and proteins associated with AR have been characterized in *A. thaliana*^16^*.* In particular, ARFs are the TFs that regulate the expression of auxin response genes^10^. Studies of *A. thaliana* and *Populus* ARF mutants have shown that ARFs play important roles in plant growth and development. For example, in *A. thaliana*, single mutations in *ARF7* or *ARF19* reduced the numbers of both lateral and adventitious roots, and the numbers of lateral and adventitious roots were even more drastically reduced in *ARF7*/*ARF19* double mutants^17^. Compared with wildtype (WT) poplars, transgenic poplars overexpressing *ARF17.1* (*Pro35s::PeARF17.1*) had numerous stems, no obvious trunk, and an increased number of ARs^18^. ARFs were strongly inhibited by members of the equally large Aux/Indole-3-Acetic Acid (Aux/IAA) protein family (29 members in *A. thaliana*) and its core inhibitor TOPLESS^19^. Key to the regulation of auxin signaling is the ubiquitin-26S proteasome system^20^-dependent disassembly of Aux/IAA proteins by a four-member family of SCF E3 ligases assembled with transport inhibitory response 1 (TIR1) F-box proteins or its relatives Auxin­Binding F-Box 1 (AFB1), AFB2, and AFB3^20^. Gretchen Hagen 3 (GH3) acyl acid amido synthase has also been reported to help regulate levels of plant hormones, including auxin^21^.

AR development is also regulated by miRNAs, including miR160, miR167, and miR396^16^. miR160, in conjunction with auxin, was shown to regulate the gene expression of *ARF10*, *ARF16,* and *ARF17*; plants overexpressing miR160c produced shorter roots^22^. miR167-*IAA-Ala Resistant3 (IAR3*) also influences lateral root development^23,24^. In cherry radish, the upregulation of miR160-*ARF16*, in conjunction with lengthier photoperiods, was shown to drive rapid root formation^25^. Using high-throughput sequencing, it was shown that the target genes of miR160 and miR390 were related to auxin signaling and involved in the formation of apple rootstock^26^. Finally, miR396 regulated the transition of root stem cells into transit-amplifying cells by interacting with growth-regulating factor (GRF) in *A. thaliana*^27^. miRNAs may also influence AR growth by targeting ARFs^10^. For example, in *A. thaliana*, miR160a, miR160b, and miR160c, which have the same mature sequence, target *ARF10*, *ARF16,* and *ARF17*, respectively^22^. By targeting ARFs, the miR160 family affects the auxin signaling pathway, and thus plays an important regulatory role in root growth and development^16^.

Despite these previous studies in model plans such as *A. thaliana* and poplar, the AR-associated functions of many miRNAs remain unknown in most horticultural tree species. To address this knowledge gap, we aimed to systematically identify candidate miRNAs that may be involved in AR development in *A. rubrum*. In addition, to investigate the AR-associated regulatory miRNA network, we aimed to characterize the expression profiles of miRNAs and their targets during *A. rubrum* root development. To this end, we constructed RNA-seq, sRNA-seq, and degradome libraries from the ARs of *A. rubrum* ‘Autumn Fantasy’. We then used bioinformatics approaches to identify AR-associated regulatory pathways, to identify TFs and miRNAs associated with those regulatory pathways, and to characterize the expression profiles of the identified miRNAs and their targets. Finally, to further explore the regulation of *ArARF10* by Ar-miR160, we overexpressed *Ar-miR160a* in *A. thaliana* and assessed produced root numbers and lengths. Our study showed that the *Ar-miR160*-*ArARF* regulatory network plays crucial roles in the growth and development of ARs. These results are of great scientific significance, as they clarify the molecular mechanism of AR formation in *A. rubrum*, as well as provide a basis for the improvement of *A. rubrum* rooting ability through molecular breeding to increase its utility in landscape applications.

## Results

### Transcriptome, sRNA, and degradome sequencing

**Figure 1:**
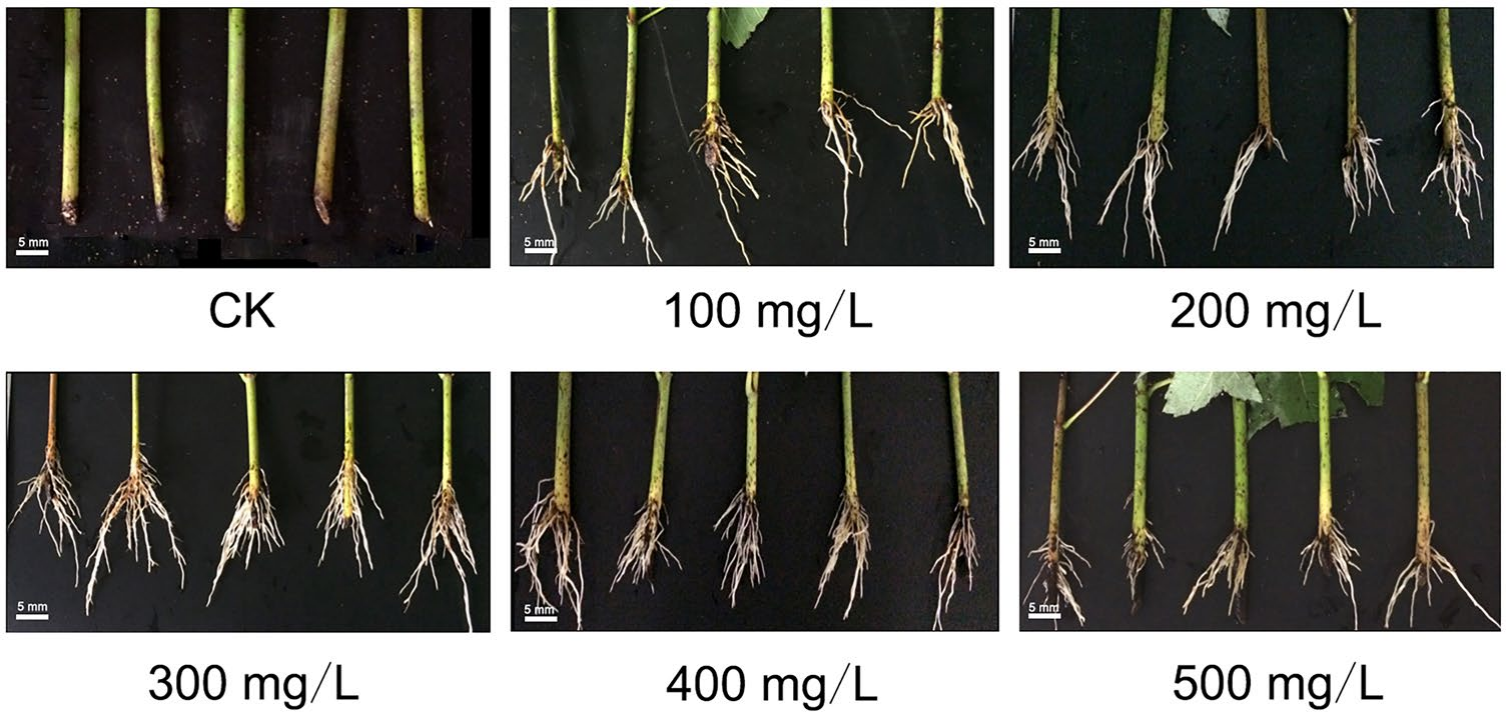
AR formation in *A. rubrum* cuttings treated with 100–500 mg/L IBA and deionized water (CK). As IBA concentration increased, the number of ARs showed a trend of increasing and then decreasing and reached a peak at IBA 300 mg/L. Scale bar, 5 mm.

After 30 days of growth, all IBA-treated groups (100–500 mg/L IBA) had developed ARs, while only a small amount of AR development was observed in the control group (Fig. 1). Importantly, substantially more ARs were developed by the cuttings treated with 300 mg/L IBA; the ARs of these cuttings were also of more consistent length and more densely packed than those formed on the cuttings treated with other concentrations of IBA (Fig. 1). Cuttings treated with 300 mg/L IBA had significantly more roots than any other cuttings. Therefore, the ARs from the cuttings treated with 300 mg/L IBA (henceforth referred to as IBA300), as well as those formed by the control cuttings, were selected for transcriptome profiling.

Illumina HiSeq sequencing generated200.4 million raw reads from the control samples and 213.29 million raw reads from IBA300. After quality filtration, 55.48 G of clean data (Q30 ≥ 93.69%) were remained. The percent GC content was greater than 38.16%, the AT and GC ratios were within a reasonable range, and there was no separation. We identified 82,468 DEGs (fold-change ≥ 2, FDR < 0.01) between the control and IBA300. Of these, 69,777 were upregulated and 12,691 were downregulated. Cross all DEG sequences, 31,385 CDS were detected, and 1417 Unigene-encoding transcription factors were predicted. These sequencing data were submitted to the SRA database with the accession numbers SRR13808891, SRR13808890, SRR13808889, and SRR13808888.

In total, approximately 51.22–52.42 million high-quality clean reads from the control and IBA300 groups were obtained using sRNA-seq. Most of the sRNAs were 21–24 nt long, with 24 nt sequences being the most common across all samples (Fig. S1). The reads remaining after the removal of tRNAs, rRNAs, snRNAs, snoRNAs, and degraded mRNAs were considered endogenous sRNAs and used in subsequent analyses. These sequencing data were submitted to the SRA database with the accession numbers SRR13808887, SRR13808886, SRR13808885 and SRR13808884.

Analysis of the sRNA-seq data showed that identified 48 known (Table S1) and 95 novel miRNAs. The length of the mature miRNAs varied from 20 to 30 nt. Due to the recognition and cleavage of DCL1 enzyme, the first base at the 5′ end of each miRNA was biased to U and resistant to G. Across all datasets, the first nucleotide of the novel miRNA was primarily biased towards U, followed by A (Fig. S2). In total, 1744 target genes cleaved by known miRNAs and 1956 miRNA-mRNA pairs were predicted using Targetfinder.

To generate a miRNA-cleaved target library (degradome) from *A. rubrum,* we first identified the mRNA transcripts targeted by miRNAs in the total RNA samples using high-throughput sequencing. We obtained 34,373,253 short reads from the control RNA samples and 28,061,482 short reads from the IBA300 RNA samples, representing the 5′ ends of uncapped, poly-adenylated RNAs. After initial processing, equal numbers of 20 and 21 nt sequence reads remained. In total, 47.66% of the unique reads were successfully mapped to the *A. rubrum* transcriptome. We identified 172 mRNAs targeted by the known miRNAs and 243 miRNA-mRNA pairs using CleaveLand (Table S2). These sequencing data were submitted to the SRA database with the accession numbers SRR13808883 and SRR13808882. Across the 172 mRNAs targeted by the known miRNAs, most were associated with plant hormone signal transduction. Of these, 80 mRNAs were either transcription factors or related to TFs, including GRFs, ARFs, SQUAMOSA promoter-binding protein-like (SPL) proteins, MYBs, scarecrow-like3 (SCL), and ethylene response factors (AP2/ERF). Consistent with the RNA-seq results, many genes were associated with the plant hormone signal transduction pathway. However, only 8.8% of the target genes were identified by degradome sequencing. This suggested that most of the predicting miRNA target genes were false positives.

**Figure 2:**
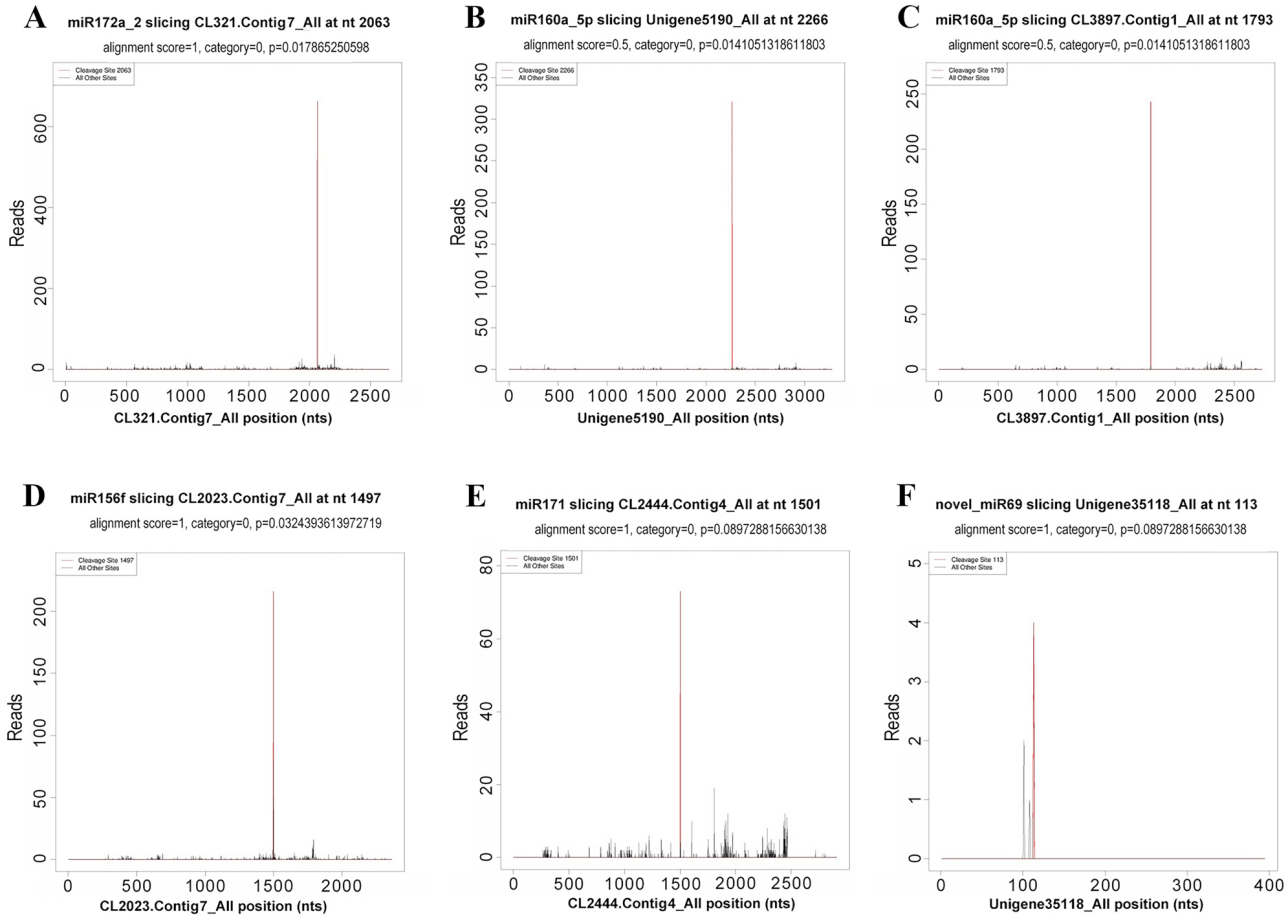
Identification of candidate miRNA targets using degradome sequencing. Six targets of five conserved and one novel miRNAs are shown in the panels as examples. The positions of the target genes are shown on the x-axis, while numbers of sequenced reads are shown on the y-axis. Each line corresponds to a degradome fragment successfully mapped to the corresponding target gene and the red lines indicate the expected miRNA positions. Category 0 indicates that, where the original data fragment was located, the abundance was equal to the maximum abundance of the mRNA, and that there was only one maximum.

The T-Plot of five conserved miRNAs and one predicted novel miRNA is shown in Fig. 2. *Ar-miR172a-2* targeted *AP2* (CL321.Contig7_All), at position 2063 nt; *Ar-miR160a-5P* targeted *ARF* (CL3897.Contig1_All), at position 1793 nt; *Ar-miR156f* targeted *SPL* (CL2023.Contig7_All), at position 1497 nt; *Ar-miR171* targeted *SCL* (CL2444.Contig4_All), at position 1501 nt. All peaks were category 0. Three of the validated differentially expressed miRNAs (*Ar-miR160a-5*, *Ar-miR171d-1*, and *Ar-miR156f*) had previously been shown to target transcription factors.

### Pathways and functions associated with the DEGs in *A. rubrum*

Across all DEGs, GO analysis indicated that 14,451 were significantly enriched in 30 GO terms between the control and IBA300 groups (27% cellular component terms, 23% molecular function terms, and 50% biological process terms; Fig. S3). In particular, the biological process terms “regulation of biological process”, “biological regulation”, “cellular process”, “metabolic process”, “signaling”, and “response to stimulus”; the cellular component terms “macromolecular complex”, “cell junction”, “nucleoid”, “organelle”, “cell part”, and “membrane part”; and the molecular function terms “binding”, “catalytic activity”, “transcription regulator activity”, and “signal transducer activity” were overrepresented in the DEGs (Fig. S3).

**Figure 3:**
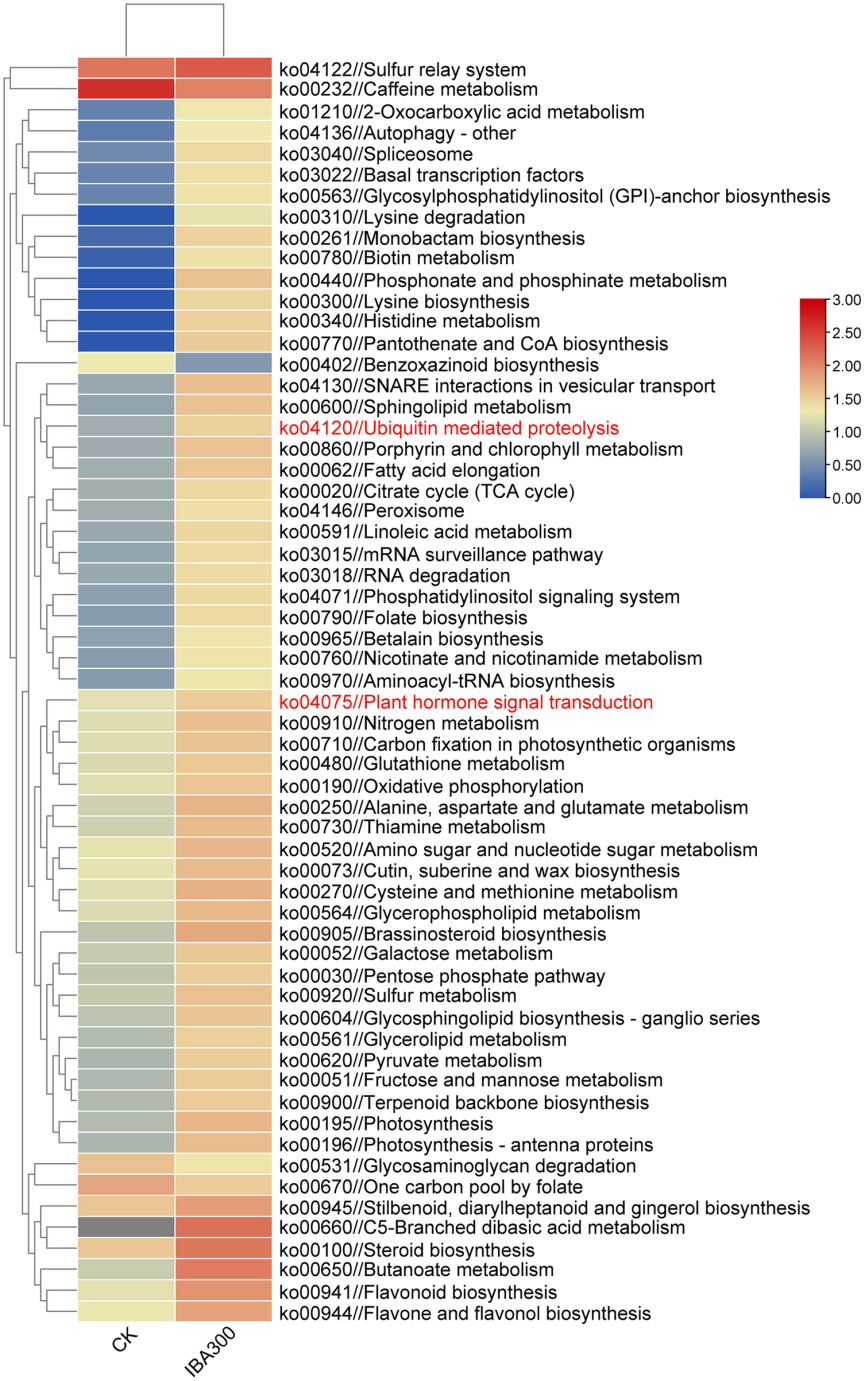
Hierarchical clustering of the differentially expressed genes associated with the major regulatory pathways. Bars represented scales of pathway expression levels (log 2), from upregulated (red) to downregulated (blue).

Of the 14,451 DEGs, 9507 were successfully mapped to the KEGG database (P-value < 0.05). KEGG mapping indicated that 59 terms and 12 metabolic pathways were significantly enriched in these DEGs, with the largest numbers of DEGs associated with the MAPK signaling pathway and the plant hormone signal transduction pathways (Fig. S4). The log-normalized FPKM values of the 60 significantly enriched metabolic pathways were used for hierarchical clustering analysis (Fig. 3). Pathways related to basal TFs, hormone transduction, and exogenous IBAs play a role in promoting gene expression. The significantly upregulated pathways were ubiquitin mediated proteolysis and hormone signal transduction (Table S4).

**Figure 4:**
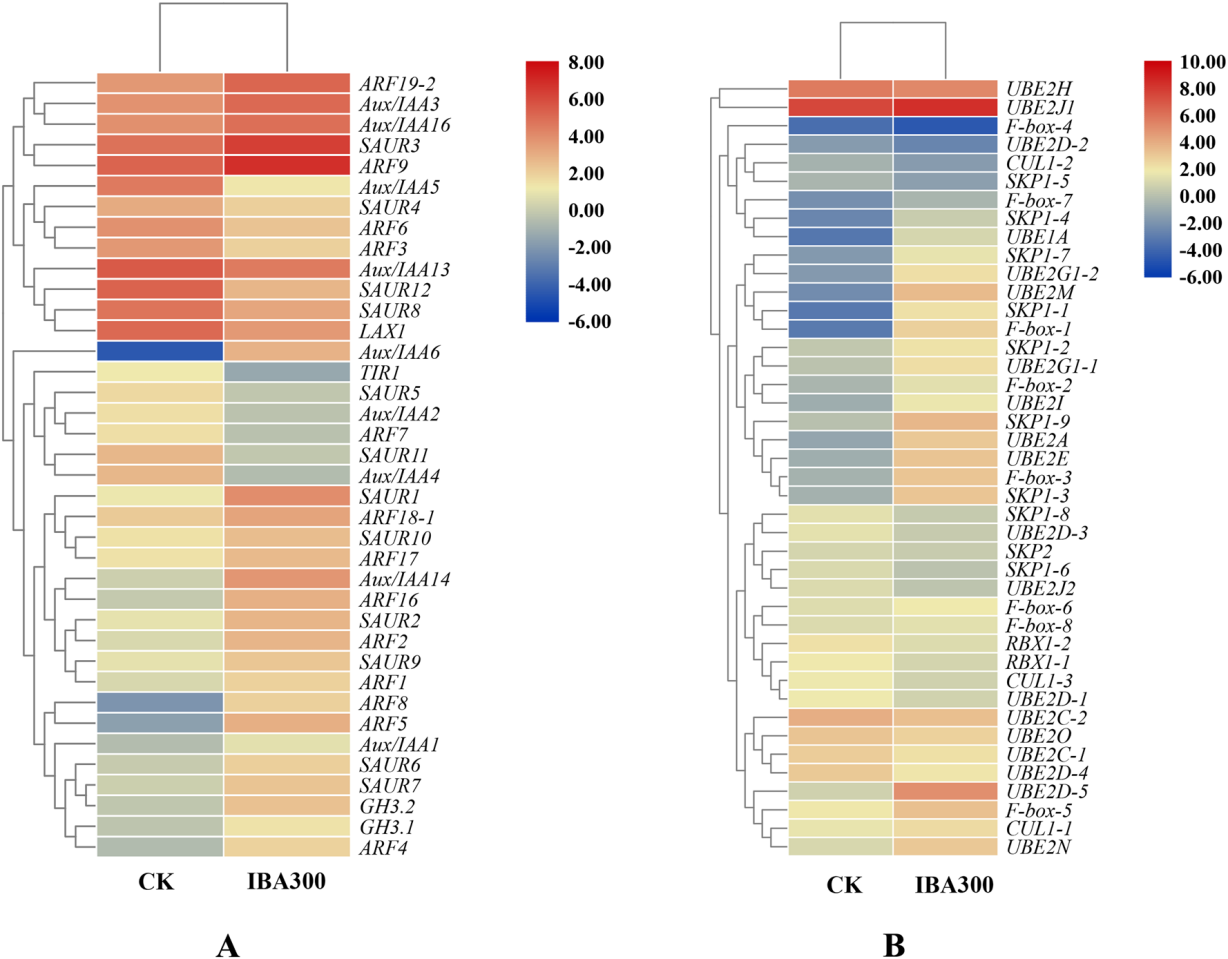
Hierarchical clustering of the differentially expressed genes in the two key pathways. (**A**) Plant hormone signaling pathway. (**B**) Ubiquitin mediated proteolysis pathway.

Many of the DEGs associated with the plant hormone signaling pathway were involved in auxin signaling transduction. For example, numerous genes in the families associated with auxin signaling (e.g., AUX, IAA, GH3, ARF, and SAUR) were significantly differentially expressed between the control group and the IBA300 group, including *Aux/IAA5* (Unigene3732_All), *SAUR11* (Unigene73966_All), *Aux/IAA4* (Unigene5767_All), *GH3* (CL4559.Contig2_All), and *ARF18-1* (Unigene5190_All). The expression levels of 24 genes (i.e., the IAAs, GH3s, ARFs, and SAURs) were increased in IBA300 as compared to the control (Fig. 4a). Our results indicated that 42 DEGs were involved in the ubiquitin-mediated proteolysis pathway (Fig. 4b). Some of these DEGs, which were associated with the mediation of Aux/IAA protein ubiquitination, were significantly upregulated, including S phase kinase-associated protein 1 (SKP1) (Unigene7049_All), cullin 1 (CUL1) (Unigene20934_All), and F-box (SCF) E3 (Unigene17129_All). The patterns of upregulation we observed suggested that the plant hormone signaling pathway and the ubiquitin mediated proteolysis pathway jointly regulate *A. rubrum* root development.

We also performed GO and KEGG analyses of the miRNA target genes. We found that the GO term “DNA binding pathway (GO: 0003677)” was significantly enriched in the target genes (p = 0.003258). KEGG pathway analysis also showed that 10 of the targets of the 220 differentially expressed miRNAs that played a significant regulatory role in the plant hormone signaling pathway belonged to three gene families (the ARF, SCL and TGA families) (Fig. S5).

### qRT‑PCR validation of the miRNAs and their targets

Nine miRNAs were selected for qRT-PCR validation. All nine miRNAs were significantly downregulated in the IBA300-treated samples as compared to the control (Fig. S6), which was consistent with our sequencing data. Thus, these nine miRNAs might play important regulatory roles in *A. rubrum* growth, especially during rooting.

**Figure 5:**
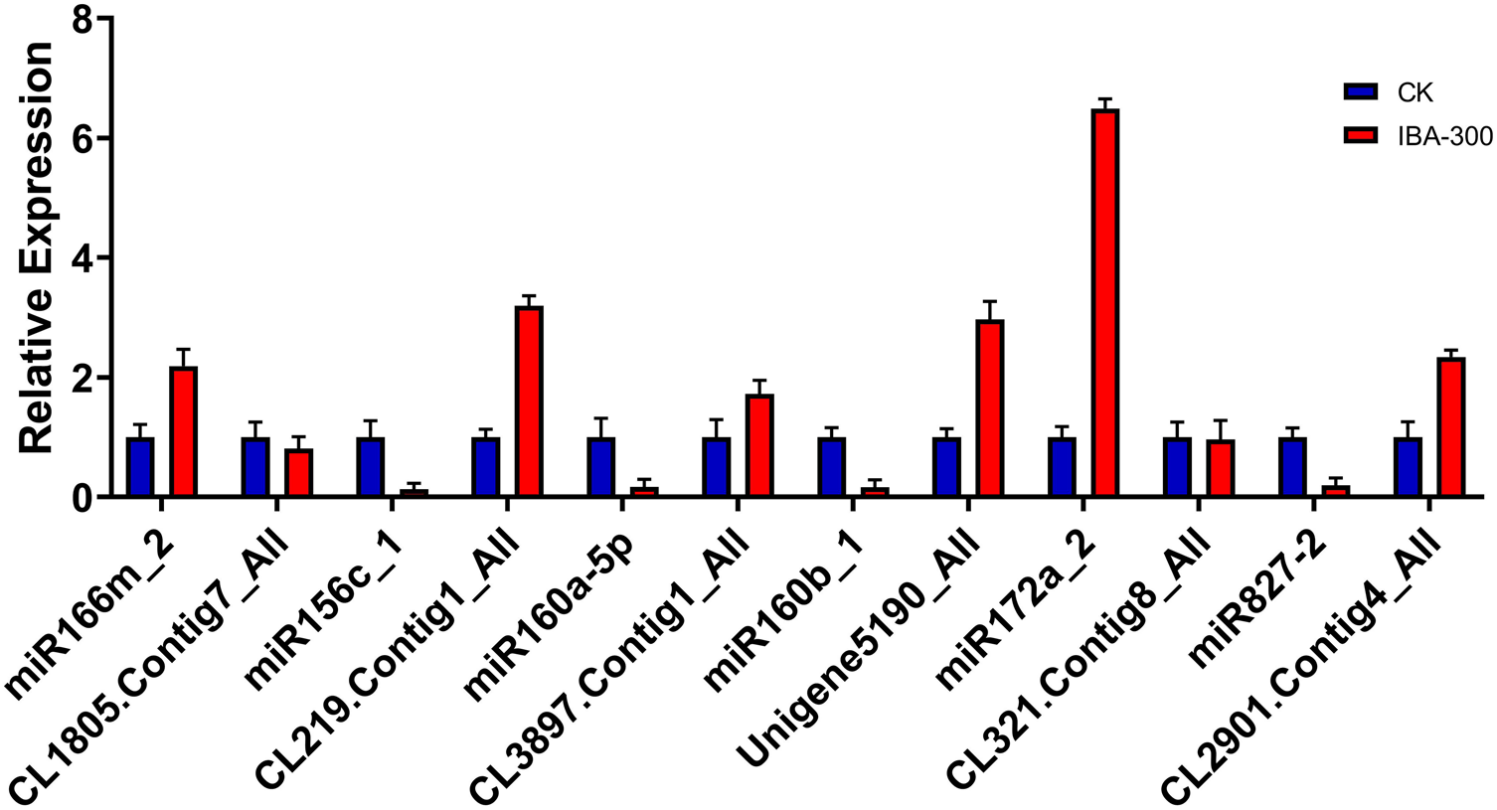
Relative expression levels of six miRNAs and target genes. *Actin6* was used as a reference for the mRNA qRT-PCR while U6 snRNA was used as a reference for the miRNA qRT-PCR. The expression in the control was set to 1.0. Mean values of three replicates are shown with standard error bars.

Next, to reveal the expression patterns of miRNA-mRNA pairs associated with plant hormone signal transduction in *A. rubrum* roots, we quantified the expression levels of six miRNAs and six target genes that were verified to interact by degradome sequencing, and where the target gene was also differentially expressed in the hormone transduction pathway. After IBA treatment, these six miRNAs showed inverse expression patterns to the corresponding six target genes (Fig. 5). This was consistent with our RNA and degradome sequencing results. Therefore, we speculated that the *Ar-miR160*-*ArARF10* interaction might be critical for the regulation of AR development in *A. rubrum*. The primers used in all qRT-PCR experiments are listed in Table S3.

### Comparative phylogenetic analysis of the ArARF protein family

Putative *ARF* genes in *A. rubrum* were identified in the RNA-seq data using BLAST. After the removal of redundant sequences and alternative splice forms, 18 ARF proteins were identified as potentially encoded by the *ArARF* genes (Table S5). In this study, we designated these proteins ArARF1–ArARF10 and ArARF16–ArARF19. We named the encoding genes as follows: *ArARF1*–*ArARF10*, *ArARF16–ArARF19, ArARF19-2*, and *ArARF19-3* (Table S5). The ORFs of the *ArARF* genes varied from 1836 bp (*ArARF10*) to 3510 bp (*ArARF19-3*). These genes encoded polypeptides of 612–1170 amino acids, with predicted molecular masses of 67.17–131.19 kDa and theoretical pIs of 5.30–8.39 (Table S5).

Our neighbor-joining (NJ) phylogenetic tree, which included 18 *ArARF*s, 22 *AtARF*s, 16 *DiARF*s, and 18 *CsARF*s, suggested that *ArARF* homologs were more common in *Dimocarpus longan* and *Citrus reticulate* than in *A. thaliana* (Fig. S7A). The *ARF* genes were clustered into three major, well-supported clades (I–III; BS > 74; Fig. S7A). Seven *ArARF* genes fell into clade Ia, four into clade Ib, one into clade IIa, three into clade IIb, and two into class III (Fig. S7A, highlighted in red). Alignment of the ARF proteins indicated that most of the ArARF proteins harbored three characteristic regions (Fig. S7C). Unsurprisingly, most of the close homologs in our phylogenetic tree shared common motifs. The three domains of ArARF proteins were comprised of a total of eight different motifs: motifs 1, 2, and 3 constituted the DNA-binding domains; motifs 6, 8, and 9 constituted the ARF domain; and motifs 5 and 10 constituted the C-terminal Aux/IAA domains. Motifs 1, 2, 3, 6, 8, and 9 were found in all 18 ArARF proteins (Fig. S7B).

### Construction of three miRNA–mRNA regulatory networks

**Figure 6:**
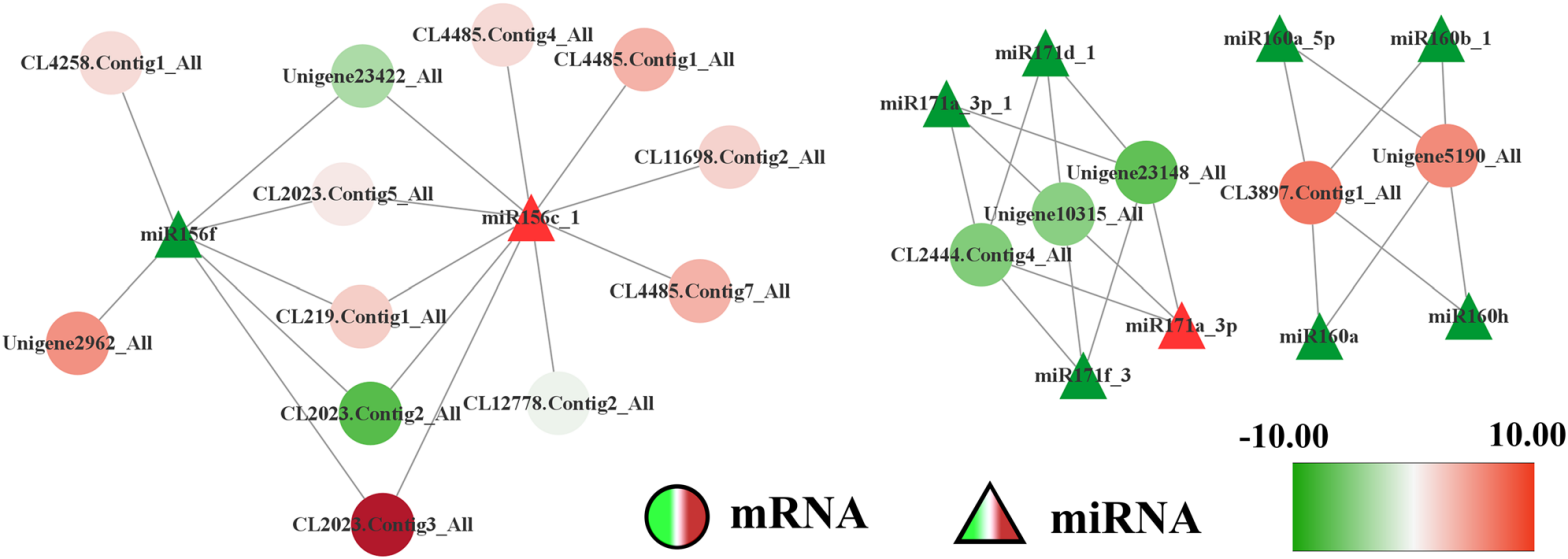
The miRNA-mRNA regulation networks of *Ar-miR160a-5p*, *Ar-miR171d-1* and *Ar-miR156f*. Upregulated genes/miRNAs are shown in red; downregulated genes/miRNAs are shown in green.

The minimum free energy (MFE) values for the precursors of our three focal miRNAs (Ar-miR160a-5p, Ar-miR171d-1, and Ar-miR156f) were − 87.10 kcal/mol, − 72.90 kcal/mol, and − 34.70 kcal/mol, respectively. The precursor sequences had typical stem-loop structures (Fig. S8). High-throughput degradome sequencing indicated that Ar-miR160a targeted *ArARF10* (CL3897.Contig1_All) and *ArARF18-1* (Unigene5190_All); Ar-miR171d-1 targeted *ArSCL6-1* (Unigene23148_All) and *ArSCL6-2* (CL2444.Contig4_All); and Ar-miR156f targeted *ArSPL-2* (CL2023.Contig1_All) (Fig. 6).

### Identification of ArARF10

Expression of fluorescent-tagged *ArARF10* in *N. benthamiana* protoplasts showed that, while the green fluorescent protein (GFP) control was dispersed throughout the cell (Fig. S9), GFP-tagged ArARF10 proteins were located in the nucleus (Fig. S9), consistent with their putative function as transcription factors. In addition, ArARF10 fused to the GAL4 DNA-binding domain activated the expression of the His-3 reporter gene in yeast (Fig. S10), indicating that this gene was a transcriptional activator.

### Overexpression of *Ar-miR160a* and *ArARF10* in *A. thaliana*

**Figure 7:**
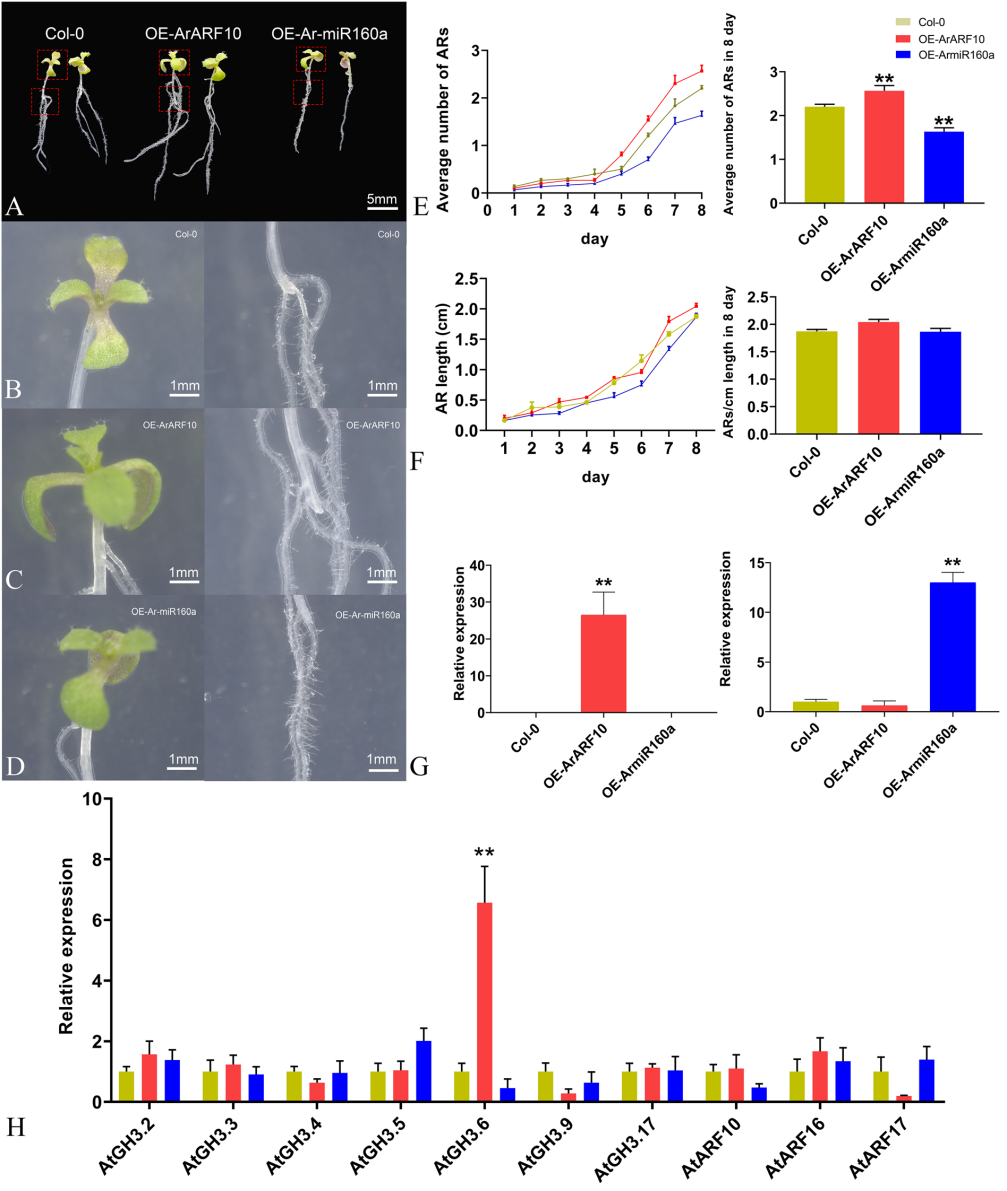
AR formation in transgenic *A. thaliana* overexpressing *ArARF10* and *Ar-miR160a*. (**A**) Comparison of transgenic plants (OE-*ArARF10* and OE-*Ar-miR160a*) to Col-0 plants. Scale bar, 5 mm. (**B**) ARs of WT phenotypes incubated under long-daylight conditions for 8 days. (**C**) Overexpression of *ArARF10* promoted AR formation. (**D**) Overexpression of *Ar-miR160a* inhibited AR formation. Scale bar, 1 mm. (**E**) Left panel shows changes in the number of ARs in Col-0, OE-*ArARF10* and OE-*Ar-miR160a* lines over 8 days. Right panel showed the number recorded on day 8, showing a significant increase in OE-*ArARF10* ARs and a significant decrease in OE-*Ar-miR160a* ARs compared to the WT. Error bars show SEM (**p < 0.01, n = 3). (**F**) Left panel shows the changes in AR length in Col-0, OE-*ArARF10* and OE-*Ar-miR160a* lines over eight days. Right panel shows the AR length recorded on day 8, with few changes in OE-*ArARF10* and OE-*Ar-miR160a* AR lengths compared to the WT. (**G**) The relative expression levels of *ArARF10* in OE-*ArARF10* (left panel) and *Ar*-*miR160a* in OE-*Ar-miR160a* (right panel). (**H**) Expression patterns of endogenous genes in transgenic and Col-0 plants. Error bars show SEM (**p < 0.01, n = 3).

GUS staining indicated that the transgenic *A. thaliana* seedlings successfully overexpressed *Ar-miR160a* or *ArARF10* (Fig. S11). Transgenic plants overexpressing *ArARF10* had many more ARs than WT plants (Fig. 7A,B,E,F), while transgenic plants overexpressing *Ar-miR160a* had fewer roots than WT plants (Fig. 7A,C–F). Consistent with this, *Ar-miR160a* and *ArARF10* were significantly more upregulated in the transgenic lines than in the WT lines (*A. thaliana* Columbia-0) (Fig. 7G). However, none of these genes were significantly differentially expressed in the transgenic plants except *AtGH3.6*, which was significantly upregulated in the transgenic plant overexpressing *ArARF10* as compared to the WT (Fig. 7H). Overexpression of *ArARF10* significantly increased the number of ARs compared with WT plants, whereas overexpression of *Ar-miR160a* had the opposite effect, indicating that this pair of target-regulated genes participates in the control of AR number.

## Discussion

The plant hormone auxin is commonly used to promote vegetative propagation in woody plants that are difficult to root^6,28^. Our results suggested that 300 mg/L IBA most effectively induced rooting in *A. rubrum* cuttings. It was found that the efficiency of AR formation was a Gaussian distribution that correlated with the concentration of IBA, indicating that, as in many other woody plants, auxin might play an important role in AR formation in *A. rubrum*.

To further investigate the molecular mechanisms regulating AR formation in *A. rubrum* in response to IBA treatment, we first identified 9507 genes that were differentially expressed in IBA-treated *A. rubrum* ARs compared to untreated *A. rubrum* ARs. KEGG pathway analysis suggested that these genes were associated with a total of 134 metabolic pathways. In particular, genes in the tryptophan metabolism, anthocyanin biosynthesis, and nitrogen metabolism pathways were generally downregulated in response to IBA treatment.

Because auxin has such a profound effect on plant growth and development, it is likely that certain checks must be in place to prevent excessive responses. Consistent with this, it has been shown that the PIN auxin efflux carrier genes transport excess auxin out of the cell via a feedback regulation loop^29^. Here, genes in the pathway responsible for synthesizing the auxin precursor tryptophan were generally downregulated after exogenous IBA treatment. Thus, we speculated that exogenous auxin might inhibit the synthesis of internal auxin in *A. rubrum*. However, the changes in expression profiles of genes in the plant hormone signal transduction pathway suggested that appropriate exogenous auxin might prolong AR development, improving rooting.

AR development is characterized by high energy requirements^30,31^. A previous transcriptomic study in carnation indicated that sucrolytic enzymatic activity was regulated at the transcript level during AR induction^32^. Consistent with this, we found that 384 of the DEGs were enriched in the “starch and sucrose metabolism” (ko00500), while several genes associated with the sucrose metabolism, including *α-glucosidase 3* (Unigene42466_All) and *glucoamylase 1* (Unigene10485_All), were significantly upregulated at the transcriptional level. This implied that the energy provided by the sucrose metabolism is crucial for the development of ARs in *A. rubrum*.

The multisubunit ubiquitin ligases of the ubiquitin–26S proteasome system (UPS), which comprise 6% of the *A. thaliana* proteome^20^, are a large and complex system of degraded proteins that play a prominent role in hormone regulation. Among the UPS, the SCF group, consisting of the Skp1, CUL1, and SCF E3 subunits^33,34^, was the most numerous and well-characterized^35,36^. Auxin mediates the binding of Aux/IAA to TIR1/AFBs, and then brings the complex to the SCF for ubiquitination and subsequent degradation^37^. Several studies have shown that auxin promotes the SCF^TIR1/AFB^ degradation of Aux/IAA proteins^38,39^. Here, most of the ubiquitination-associated DEGs were implicated in the ubiquitination and degradation of Aux/IAA by the SKP1-CUL1-SCF complex, mediated by auxin. In particular, genes that mediate the ubiquitination of Aux/IAA proteins, including SKP1 (Unigene7049_All), CUL1 (Unigene20934_All), and SCF E3 F-box (Unigene17129_All), were significantly upregulated in response to exogenous auxin treatment. This result suggested that auxin-mediation ubiquitination and degradation of Aux/IAA might facilitate AR formation in *A. rubrum*.

Members of the *Aux/IAA* family are short-lived nuclear proteins that play a critical role in suppressing the expression of ARF-activated genes^19,40^. Aux/IAA proteins have been known to bind to ARFs and prevent the activation of auxin-responsive genes in the absence of auxin^41^. At high levels of auxin, Aux/IAA proteins are ubiquitinated by interaction with the TRANSPORT INHIBITOR RESPONSE 1/AUXIN SIGNALING F-BOX (TIR1/AFB) (Unigene20637_All) receptor and subsequently degraded by the 26S proteasome^42,43^. The SCF^TIR1/AFB^-Aux/IAA-ARF signaling system thus allows for a diversity of auxin responses. Interestingly, our transcriptome sequencing results indicated that this signaling regulatory system in *A. rubrum* ARs was significantly affected by treatment with exogenous auxin. Altering the transcription of genes in the auxin signaling pathway might be a genetic strategy used by *A. rubrum* in response to auxin alterations. Analysis of these genes might help to identify those that play a key role in AR development.

We identified 14 putative *ArARF* genes in the AR transcriptome of *A. rubrum*, most of which contained an amino-terminal DNA binding domain, a middle region that functioned as the activation or repression domain, and a carboxy-terminal dimerization domain. Four of these genes were significantly upregulated in response to IBA treatment: *ArARF5* (CL11296.Contig1_All), *ArARF17* (CL5608.Contig3_All), *ArARF18-1* (Unigene5190_All), and *ArARF19-1* (CL1642.Contig1_All). The nuclear targeting of the ArARF10 protein was consistent with transcriptional regulation and transcriptional activation functions. Structural domain function analysis implied that ArARF10 promoted AR development in *A. rubrum* by activating downstream auxin-responsive genes. These results indicated that ArARFs might play different roles during AR development in *A. rubrum* cuttings.

Phylogenetic analysis of the ARF proteins from *A. rubrum*, Longan, *Citrus* sp., and *A. thaliana* suggested that ARF proteins fell into three major clades, consistent with previous evolutionary analyses^44–47^. Consistent with this, previous studies have shown that ARF proteins include a highly conserved region of about 100 amino acid residues in the N-terminal region, corresponding to the DNA-binding domains^48^. Motif analysis identified 10 unique motifs across the 18 ArARF proteins^49^. Notably, ArARF8, ArARF10, and ArARF6-2 exhibited a high degree of homology with DiARF8, DiARF1, CsARF6, CsARF8, and CsARF10*.* In addition to the high degree of domain conservation and homology in ARF proteins among species, numbers of ARF copies are similar between *A. rubrum* and other species in the Sapindales, including Longan, and *Citrus* sp.^44,50^. This indicated that species in this order might undergo similar whole-genome duplications, and it was speculated that ARF diversification might represent an adaptation to environmental changes^51^.

Although ARF TFs have been shown to play a critical role in the regulation of root development and auxin correspondence^52^, miRNA interactions with TFs and other targets might also influence the regulatory networks that control the root transcriptome and might play a key role in the eventual translation of mRNAs^53^. Using sRNA sequencing, we identified six differentially expressed miRNAs associated with plant hormone signal transduction. *miR171*, *SCL6-II*, *SCL6-III*, and *SCL6-IV* are expressed ubiquitously in plants^54,55^. Transgenic plants overexpressing *MIR171c* (*35Spro–MIR171c*) and *scl6-II scl6-III scl6-IV* triple mutant plants exhibited pleiotropic phenotypes^56^, including alterations in shoot branching, plant height, chlorophyll accumulation, root development, flower architecture, and leaf shape and pattern. We found that Ar-miR171 cleaved *ArSCL* in *A. rubrum.* Ar-miR171 was significantly downregulated after exogenous IBA treatment, suggesting that the expression of miR171/*SCL* might be related to auxin activity in *A.rubrum*. miR171/*SCL* may play an important role in AR development. In *Arabidopsis*, *SPL* was targeted by miR156, while *SPL3*, *SPL9* and *SPL10* were involved in suppressing lateral root growth^57^. In addition, both *miR156* and *SPLs* were sensitive to auxin signaling^58^. In our work, we found that Ar-miR156 was sensitive to exogenous IBA and targeted *ArSPL*, suggesting that miR156/*SPL* might be involved in AR development in *A. rubrum*.

Ar-miR160a-5p and Ar-miR160b-1 targeted and cleaved *ArARF10* and *ArARF18-1*. *Ar-miR160* and its target gene *ArARF10* showed opposite expression patterns during AR formation in the presence of exogenous auxin. Most miRNAs in the *miR160* family regulate plant development through the targeted cleavage of ARFs. Transgenic *A. thaliana* and *Medicago truncatula* overexpressing *miR160* had shorter roots than their WT counterparts^22,59^, and a study in Longan showed that *Dlo-miR160* negatively regulated *DiARF10*, *DiARF16*, and *DiARF17* to affect hormone signaling and somatic embryogenesis^60^. In developing ARs of poplars, peu-miR160a was downregulated, while its target (*peARF17.1*) was upregulated^61^; AR formation in transgenic poplar overexpressing peu-miR160a was inhibited, whereas AR formation in polar overexpressing *PeARF17.1* or *PeARF17.2* was promoted^18^. Finally, *A. thaliana* overexpressing *miR160* exhibited reduced *AtARF10* and *AtARF16* expression, shortened roots, and abnormal root cap development, as well as uncontrolled cell division and root apical meristem (RAM) differentiation^22^. Considering the highly conserved nature of the *miR160* family^62^, we speculated that *Ar-miR160* plays an important role in AR development through the targeted cleavage of *ARF* genes.

Despite this evidence supporting the important role of *miR160* in AR development, the specific effects of *miR160* expression tend to vary among species. For example, miR160 overexpression in poplar suppressed AR development and elongation, but overexpression of *miR160a* in *A. thaliana* promoted AR development^16^. Here, *A. rubrum* seedlings overexpressing *Ar-miR160a* generally had fewer ARs than the WT. Similar results were observed in poplar seedlings overexpressing *Peu-miR160a*^18^.

**Figure 8:**
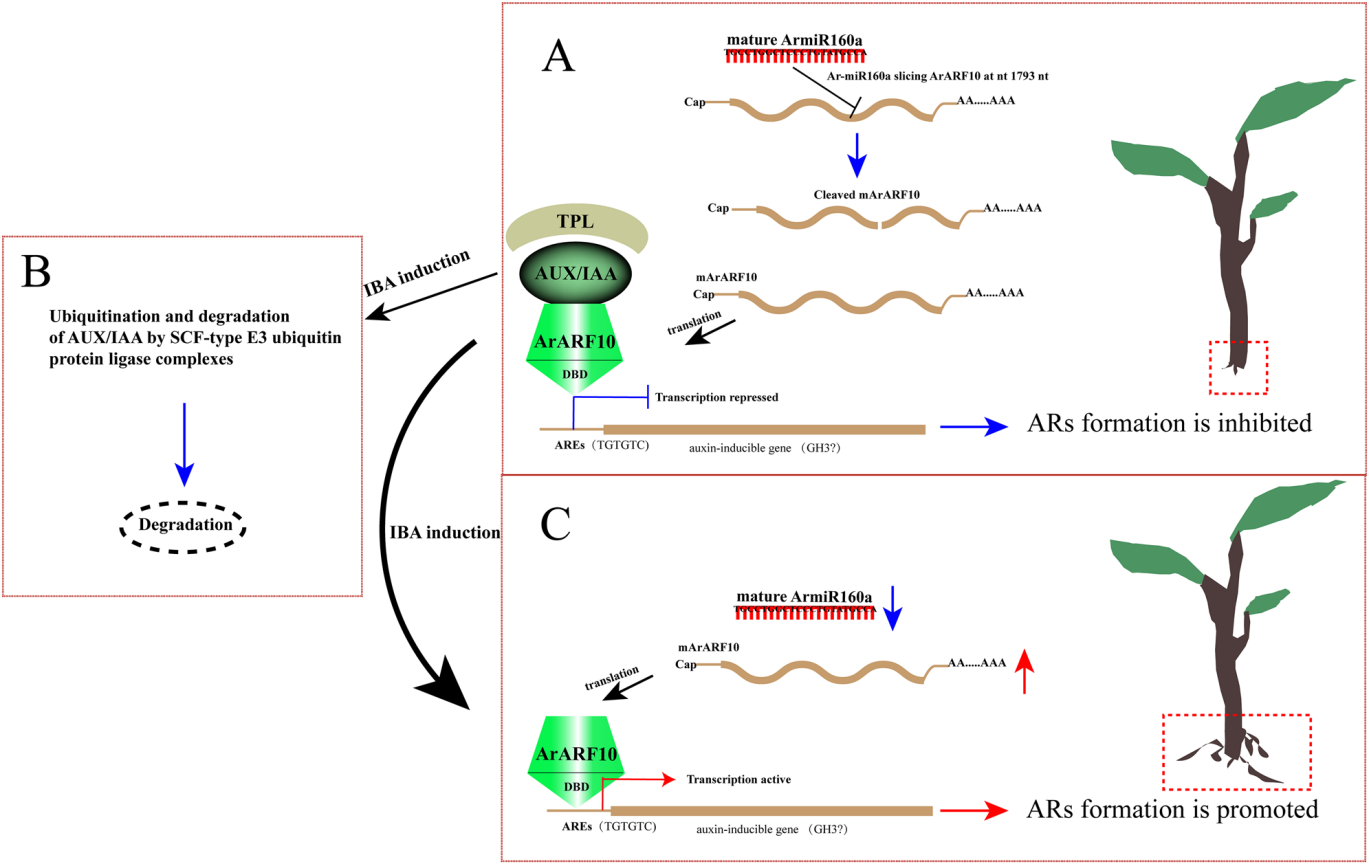
Schematic presentation of the regulation of AR growth in *A. rubrum*. (**A**) Under low auxin conditions, Ar-miR160 specifically cleaves mRNA-*ArARF10* to repress *ArARF10* expression, while Aux/IAA prevents auxin-inducible gene transcription by recruiting TPL to co-inhibit the binding of ArARF10 AREs in the auxin-inducible gene promoter. (**B**) Under increased levels of exogenous auxin, Aux/IAA is ubiquitinated and degraded by the SCF-type E3 ubiquitin protein ligase complex, and ArARF10 binds to AREs in the promoters of auxin-inducible genes to increase transcription. (**C**) The downregulation of *Ar-miR160* also caused the translation of additional mRNA-*ArARF10* into protein, as well as additional downstream functions that promoted AR formation.

Determination of target gene function may help to clarify miRNA function. Transgenic *A. thaliana* overexpressing *ArARF10* had more ARs than the WT; *GH3.6* expression was also significantly upregulated in the transgenic plants. *GH3* genes are highly responsive to exogenous auxin^63^; auxin stimulates AR development by inducing the expression of the *GH3.3*, *GH3.5*, and *GH3.6* genes via the positive regulators *ARF6* and *ARF8*. Consistent with this, the three-null mutant *gh3.3gh3.5gh3.6* had fewer ARs than its wild-type counterpart^64^. These results suggested that, in *A. thaliana*, high levels of auxin led to the overexpression of *ArARF10*, stimulating AR formation. Interestingly, although *ArARF10* was functionally similar to *AtARF8* and *AtARF6*, *ArARF10* overexpression did not upregulate *GH3.3* and *GH3.5*, implying that auxin regulation pathways might differ between *A. rubrum* and *A. thaliana*. Our examination of *Ar-miR160*-*ArARF10* function in AR development in *A. rubrum* revealed differences with its role in AR development in *A. thaliana*. *AtARF17* suppresses AR development in *A. thaliana*^16^, whereas *ArARF10* played a positive role in AR development in the model. Interestingly, this difference was also present between *Populus* and *A. thaliana*. *PeARF17.1* plays a similar role to *ArARF10*^18^.In this study, we identified two pathways involved in the auxin response and analyzed the *ArARF* genes in those pathways. We characterized the TF *ArARF10*, and separately verified the role of the *Ar-miR160a*-*ArARF10* interaction in AR development. We found that, during AR development, *Ar-miR160* acted as a negative regulator to cleave the positive regulator *ArARF10*; this process was also integrated into the plant hormone signaling pathway and the ubiquitin mediated proteolysis pathway. Based on our results, we propose a model clarifying the mechanisms underlying the promotion of AR growth by exogenous auxin (Fig. 8): under low auxin conditions, Aux/IAA co-inhibits ARF by recruiting the OPLESS (TPL) family and preventing the ARF activation of downstream auxin-inducible genes, regulating AR formation at the protein level^65,66^. Simultaneously, Ar-miR160 suppresses *ArARF10* expression by specifically cleaving *ArARF10* mRNA, thus regulating AR formation at the transcriptional level (Fig. 8A). When exogenous auxin levels are elevated, Aux/IAA is ubiquitinated and degraded by the SCF-type E3 ubiquitin protein ligase complex, and ARF binds to AREs in the promoters of the auxin-inducible genes to promote transcription (Fig. 8B)^20^. The downregulation of *Ar-miR160* also implies that more *ArARF10* mRNAs are translated into proteins and perform downstream functions (Fig. 8C). In this way, *A. rubrum* responds sensitively and effectively to exogenous auxin during AR formation, leading to increased AR production. Thus, our work provides new insights into the key regulatory roles played by this module in the AR development of *A. rubrum*. These results are of great scientific significance, as they clarify the molecular mechanism of AR formation in *A. rubrum*, as well as provide a basis for the improvement of *A. rubrum* rooting ability through molecular breeding to increase its utility in landscape applications.

## Conclusion

In this study, AR development in *A. rubrum* was significantly increased by treatment with 300 mg/L IBA. Changes in gene expression after exogenous auxin treatment were explored using high-throughput sequencing. We found that two key regulatory pathways (ubiquitin mediated proteolysis and plant hormone signal transduction), the miRNA *Ar-miR160a,* and its target gene *ArARF10*, were involved in the auxin response. Experiments overexpressing *ArARF10* or *Ar-miR160a* showed that *ArARF10* increased AR development, while *Ar-miR160a* inhibited AR development. Our results may help to clarify the key regulatory pathways and genes associated with AR formation in woody plants, and provide a basis for the eventual improvement of *A. rubrum* rooting success.

## Methods

### The use of plant materials and ethical approval statement

This study including sample collection was conducted following China’s Biodiversity Conservation Strategy and Action Plan (2011–2030) (Index number: 000014672/2010-00714) and the Seed Law of the People’s Republic of China (2015 Revised Version), which together permit use of biological resources by Chinese researchers for scientific purposes.

### Plant materials

We collected full and semi-woody annual branches (60 cm in length) from *A. rubrum* hybrid ‘Autumn Fantasy’ trees. The branches were divided into 8–10 cm cuttings, each containing two nodes. Cuttings were disinfected with 0.5% potassium permanganate solution, and then soaked in 100 mg/L, 200 mg/L, 300 mg/L, 400 mg/L, or 500 mg/L IBA for 1 h to induce AR formation (n = 50 branches per group). The depth of each cutting was 3 cm. Control cuttings were soaked in clean water for one hour (n = 50). Cuttings were then grown in a greenhouse under a 16 h light (25 °C)/8 h dark (18 °C) photoperiod. After 30 days of growth, roots were washed and the numbers of ARs were recorded. The ARs from branches soaked in 300 mg/L IBA and in clear water were stored at − 80 °C for RNA sequencing (RNA-seq), small RNA sequencing (sRNA-seq), and degradome sequencing.

### RNA isolation, library construction, and sequencing

Total RNA was isolated from the CK and IBA300 groups using Trizol Reagent (Invitrogen, Carlsbad, CA, USA) following the manufacturer’s instructions. After verification of RNA sample quality using a Nanodrop 2000 (Thermo Fisher Scientific, Waltham, MA, USA), mRNA was reverse transcribed using PrimeScriptTM (Takara, China). Finally, the double-stranded cDNA was purified using a DNA purification kit (QIAGEN, Germany) to generate high quality cDNA. Libraries were prepared from 300 to 500 bp size-selected fractions following adapter ligation and agarose gel separation. The libraries were sequenced using a paired-end read protocol with 100 bp of data collected per run on the Illumina HiSeq 2000.

The sRNA libraries were constructed using a NEBNext Multiplex Small RNA Library Prep Set for Illumina and sequenced on a BGISEQ-500 system. mRNA was captured using magnetic beads. Mixed biotinylated random primers and mRNA were used for reverse transcription. After library preparation was completed, the constructed library was sequenced using an Illumina HiSeq2500, with a single-ended read length of 1 × 50 bp.

### Bioinformatics analysis of the sequencing data

The raw transcriptome sequencing data were filtered by removing adapters and low quality sequences. The clean data were assembled using Trinity version 2.0.6^67^. The transcripts were clustered and redundant transcripts were eliminated using Tgicl to obtain Unigenes^68^. The assembled transcripts were quality-checked by comparison with conserved genes using the single-copy direct homology database BUSCO^69^. Potential duplicate molecules were removed from the aligned BAM format records. Fragments per kilobase of exon per million fragments mapped (FPKM) values were used determined relative gene expression levels in Cufflinks^70^. Novel genes were identified based the transcripts newly discovered using Cufflinks. Genes encoding peptides with less than 50 amino acid residues were filtered. DEGs were detected using DEGseq; gens were considered differentially expressed when thefold change in relative expression was ≥ 2 and the Q-value was ≤ 0.001^71^.

The raw sRNA sequencing data were filtered by removing adapters and low-quality data to generate clean reads. Unique sRNAs were aligned to the miRNA precursors of corresponding species in miRBase to obtain a miRNA count. We used miRA, which identifies miRNAs based on the ability of the miRNA precursors to form hairpin secondary structures, with default plant parametersto predict novel miRNAs^72^. Fold-change in miRNA expression levels were calculated using Transcripts Per Kilobase Millions (TPMs)^73^. DEGseq was used to identify differentially expressed miRNAs between IBA300 and CK^71^. The P-values calculated for each gene were adjusted to Q-values to correct for multiple testing using two alternative strategies^74^. miRNAs were considered differentially when fold change was ≥ 2 and Q-value was ≤ 0.001.

Standard sequences were compared to the degradome sequences using reads per million (RPM) to remove redundancy. The Needle program in the EMBOSS package was applied to derive all sequences that matched the sequences in the previous sRNA library, and then the columns were scored according to the plant miRNA/target pairing criteria^75,76^. The mRNA sequences of target genes paired with the sRNA sequences of *A. rubrum* were predicted by Targetfinder, with a prediction score cutoff of four. The target genes corresponding to the predicted miRNAs and the mRNAs in the degradome were combined and analyzed to identify shared mRNAs targeted by the miRNAs. Degradome peak classifications and scores, as well as the prediction results, were plotted^77^. The MFE values for these precursors were predicted using the classic algorithm of Zuker and Stiegler^78^.

### GO and KEGG enrichment of the differentially expressed genes (DEGs)

DEGs were annotated by searching the sequences against the NR, Swiss-Prot, GO, KEGG databases using BLAST^79–81^. We investigated the GO enrichment of DEGs using the Gene Ontology Consortium (http://geneontology.org/); p-values were FDR corrected using DEGseq^71^, and terms with Q-value ≤ 0.05 were considered significantly enriched in the DEGs. We identified KEGG pathways significantly associated with the DEGs based on relative expression levels. Hierarchical clustering of expression data was performed using TBtools^82^.

### Identification and classification of *ARF* genes

We searched 23 protein sequences from *A. thaliana* (https://www.arabidopsis.org/) against the transcript of *A. rubrum* using Tblastn, with the following settings: matrix, BLOSUM62; expect value, < 1e − 005; gap-existence, 11; gap-existension extension, 1; and filter, low-complexity. Previously published *ARFs* from *A. thaliana*, *C. sinensis*, and *D. longan* were identified using a BLASTP search with the score value set to ≥ 100 and e-value set to ≤ e^−10^ ^45,83^. Next, the Pfam database (http://pfam.xfam.org/) was used to determine whether each candidate ARF sequence belonged to the ARF gene family^84^. To exclude overlapping genes, all candidate ARF genes were aligned with ClustalW and checked manually. The SMART (http://smart.embl-heidelberg.de/) and InterProScan (http://www.ebi.ac.uk/Tools/pfa/iprscan/) web servers were used to examine the conserved domains of the identified *A. rubrum* genes. Obtained *ArARF* genes were named based on their *A. thaliana* homologs. Protein characteristics, including molecular weight (MW), isoelectric point (pI), and length, were predicted using the online ProtParam tool.

### Phylogenetic and conserved motif analysis of the ArARFs

To explore inter- and intra-specific phylogenetic relationships among ARF proteins, we constructed a dataset of ARF proteins including the ArARFs identified in this study, as well as 22 *A. thaliana* ARFs, 16 *D. longan* ARFs, and 18 citrus ARFs (referred to herein as *AtARF*, *DiARF*, and *CsARF* respectively). Neighbor-joining (NJ) phylogenetic analysis of the 74 ARF protein sequences was performed using MEGA7^85^, with 1000 bootstrap (BS) replicates. The best-fit model of nucleotide substitution (i.e., that with the lowest BIC score) was JTT + G + I. All positions with less than 80% site coverage were eliminated; that is, fewer than 20% alignment gaps, missing data, and ambiguous bases were allowed at any position^86^. To investigate the structural differences among *ArARF* genes, conserved motifs in the predicted ARF proteins were investigated using online MEME analysis (http://meme.nbcr.net/meme/), with an optimum motif width of 6–50. The maximum number of motifs was set to 10^87^. Results were visualized using TBtools^88^.

### Quantitative reverse-transcription PCR (qRT-PCR) assay

qRT-PCRs were performed using a CFX Connect TM Real-Time System. Each total RNA sample (2 μg) was digested with RNase-free DNase I (Invitrogen) to remove genomic DNA contamination and subsequently reverse transcribed into cDNA using an Oligo (dT)18 primer. For the qRT-PCR of the miRNAs, U6 snRNA was used as the internal control. For the qRT-PCR analyses of the miRNA target genes^89^, *Actin6* was used as the internal control^90^. PCR volumes (20 μl) contained 1 μl of 20× diluted cDNA, 5× SYBR buffer, and 0.25 μM forward and reverse primers; the gene-specific primers used for qRT-PCR are given in Table S3. The thermal cycling conditions were as follows: 95 °C for 30 s, followed by 40 cycles of 5 s at 95 °C and 30 s at 60 °C. All reactions were performed in triplicate in three independent experiments. The relative fold-changes in miRNA and gene expression levels were calculated using the 2^−ΔΔCt^ method while the relative fold-change in *ArARF10* expression was calculated using the 2^−ΔCt^ method^91^.

### Subcellular localization and transcriptional activation

The full-length cDNA sequences of the target gene *ArARF10* were cloned from the cDNA of ARs from *A. rubrum*. The subcellular location of ArARF10 was determined by transfecting GFP-tagged ArARF10 into 3–4-week-old *Nicotiana benthamiana* protoplasts. The full-length cDNA of *ArARF10* was fused in-frame with GFP cDNA and ligated between the CaMV 35S promoter and the nopaline synthase terminator. The fluorescence signals in the tobacco protoplasts were examined under a confocal laser scanning microscope (Axio Lab A1)^92^. The transcriptional activation of ArARF10 was investigated by transforming the pGBKT7 construct (in which ArAFR10 was fused with the GAL4 DNA-binding domain) into yeast strain AH109. Yeast strain AH109 contains the His-3 reporter genes. The transformants were streaked on the SD/Trp- and SD/Trp-/His-media. After incubation at 28 °C for 3 days, the growth of the transformants was evaluated. The transformed yeast cells were grown on synthetic defined (SD) media (with or without His); yeast with active His-3 reporter genes were able to grow on synthetic defective plates.

### Constructs and plant transformation

We next tested whether the overexpression of *Ar-miR160a* and *ArARF10* affected the growth and development of *A. thaliana.* The *Ar-miR160a-5p* precursor was cloned from *A. rubrum* genomic DNA. The precursor sequence of *Ar-miR160a-5p* and the ORFs of *ArARF10* were cloned into the entry vector pTOPO-T. After verification by sequencing, the fragments of the *Ar-miR160a-5p* precursor and *ArARF10* inserted into the entry vector were transferred to the destination vector pCAMBIA1301 via an LR reaction. The constructed vectors (*Pro35S::Ar-miR160a* and *Pro35S::ArARF10*) were driven by the CaMV 35S promoter. The pCAMBIA1301 vector contained a GUS reporter gene initiated by the 35S promoter.

The constructs (*Pro35S::Ar-miR160a* and *Pro35S::ArARF10*) were introduced into WT *A. thaliana* (Columbia-0) using electroporation and *Agrobacterium*-mediated transformation. Seeds of the plants exhibiting transgenic phenotypes were sterilized and sown on 1/2 Murashige and Skoog (1/2 MS) medium, as described previously^93^. Plates were incubated at 4 °C for 48 h for stratification and then exposed to light for several hours to induce germination. Plates were next wrapped in three layers of aluminum foil and placed in the dark until the seedlings had an average hypocotyl length of 6 mm (48 h). The *A. thaliana* primordium was excised using a sterile blade, preserving about 6 mm of the hypocotyl, and then the seedlings were exposed to a natural photoperiod (16 h light/8 h dark) to induce AR formation. AR lengths and numbers were measured using ImageJ^94^ 8 days after transfer to lighted conditions. For each biological replicate, at least ten seedlings were analyzed, and each experiment was repeated at least three times. One-way ANOVAs, combined with Tukey's multiple comparison tests, were performed using GraphPad Prism 8 to analyze differences in means and variances among genotypes.

### GUS staining

After eight days of cultivation, transgenic and WT *A. thaliana* seedlings were washed and completely immersed in GUS staining solution (Coolaber, China). After overnight staining at 37 °C in the dark, seedlings were decolorized in 70% ethanol, examined, and photographed.

## Supplementary Information

Supplementary Information.

## Data Availability

The sequencing data were submitted to the SRA database with the accession numbers SRR13808891, SRR13808890, SRR13808889, SRR13808888, SRR13808887, SRR13808886, SRR13808885 and SRR13808884.The data that support the conclusions are within this article and its additional files. All data and plant materials used in current study are available from the corresponding author on reasonable request.

## References

[1] Abrams MD (1998). The red maple paradox. Bioscience.

[2] Lahr EC, Dunn RR, Frank SD (2018). Variation in photosynthesis and stomatal conductance among red maple (Acer rubrum) urban planted cultivars and wildtype trees in the southeastern United States. PLoS ONE.

[3] Pijut PM, Woeste KE, Michler CH (2011). 6 promotion of adventitious root formation of difficult-to-root hardwood tree species. Hortic. Rev..

[4] Sibley JL, Joseph Eakes D, Gilliam CH, Keever GJ, Dozier WA (1995). Growth and fall color of red maple selections in the Southeastern United States. J. Environ. Horticult..

[5] Druege U, Franken P, Hajirezaei MR (2016). Plant hormone homeostasis, signaling, and function during adventitious root formation in cuttings. Front. Plant Sci..

[6] Geiss, G., Gutierrez, L. & Bellini, C. Adventitious root formation: New insights and perspectives. *Annu. Plant Rev. Online*. 127–156 (2018).

[7] Druege U (2019). Molecular and physiological control of adventitious rooting in cuttings: Phytohormone action meets resource allocation. Ann. Bot..

[8] Pacurar DI, Perrone I, Bellini C (2014). Auxin is a central player in the hormone cross-talks that control adventitious rooting. Physiol. Plant..

[9] Sablowski R (2011). Plant stem cell niches: From signalling to execution. Curr. Opin. Plant Biol..

[10] Guilfoyle TJ, Hagen G (2007). Auxin response factors. Curr. Opin. Plant Biol..

[11] Epstein E, Ludwig-Müller J (1993). Indole-3-butyric acid in plants: Occurrence, synthesis, metabolism and transport. Physiol. Plant..

[12] Simon S, Petrášek J (2011). Why plants need more than one type of auxin. Plant Sci..

[13] Lakehal A, Bellini C (2019). Control of adventitious root formation: Insights into synergistic and antagonistic hormonal interactions. Physiol. Plant..

[14] Legué V, Rigal A, Bhalerao RP (2014). Adventitious root formation in tree species: Involvement of transcription factors. Physiol. Plant..

[15] Rigal A (2012). The AINTEGUMENTA LIKE1 homeotic transcription factor PtAIL1 controls the formation of adventitious root primordia in poplar. Plant Physiol..

[16] Gutierrez L (2009). Phenotypic plasticity of adventitious rooting in Arabidopsis is controlled by complex regulation of AUXIN RESPONSE FACTOR transcripts and microRNA abundance. Plant Cell.

[17] Okushima Y, Fukaki H, Onoda M, Theologis A, Tasaka M (2007). ARF7 and ARF19 regulate lateral root formation via direct activation of LBD/ASL genes in Arabidopsis. Plant Cell.

[18] Liu S (2020). The peu-miR160a–PeARF17.1/PeARF17.2 module participates in the adventitious root development of poplar. Plant Biotechnol. J..

[19] Luo J, Zhou J-J, Zhang J-Z (2018). Aux/IAA gene family in plants: Molecular structure, regulation, and function. Int. J. Mol. Sci..

[20] Vierstra RD (2009). The ubiquitin-26S proteasome system at the nexus of plant biology. Nat. Rev. Mol. Cell Biol..

[21] Westfall, C.S., Sherp, A.M., Zubieta, C., Alvarez, S. & Jez, J.M. Arabidopsis thaliana GH3.5 acyl acid amido synthetase mediates metabolic crosstalk in auxin and salicylic acid homeostasis. *Proc. Natl. Acad. Sci. USA*. **113** (2017). 10.1073/pnas.1612635113 PMC5137743 27849615

[22] Wang J-W (2005). Control of root cap formation by microRNA-targeted auxin response factors in Arabidopsis. Plant Cell.

[23] Kinoshita N (2012). IAA-Ala Resistant3, an evolutionarily conserved target of miR167, mediates Arabidopsis root architecture changes during high osmotic stress. Plant Cell.

[24] Olatunji D, Geelen D, Verstraeten I (2017). Control of endogenous auxin levels in plant root development. Int. J. Mol. Sci..

[25] Guo R (2019). Effect of photoperiod on the formation of cherry radish root. Sci. Hortic..

[26] Li K (2019). miRNAs associated with auxin signaling, stress response, and cellular activities mediate adventitious root formation in apple rootstocks. Plant Physiol. Biochem..

[27] Rodriguez RE (2015). MicroRNA miR396 regulates the switch between stem cells and transit-amplifying cells in Arabidopsis roots. Plant Cell.

[28] Yu J, Liu W, Liu J, Qin P, Xu L (2017). Auxin control of root organogenesis from callus in tissue culture. Front. Plant Sci..

[29] Weijers D, Wagner D (2016). Transcriptional responses to the auxin hormone. Annu. Rev. Plant Biol..

[30] Agulló-Antón MÁ, Sánchez-Bravo J, Acosta M, Druege U (2011). Auxins or sugars: What makes the difference in the adventitious rooting of stored carnation cuttings?. J. Plant Growth Regul..

[31] Agulló-Antón MÁ (2014). Early steps of adventitious rooting: Morphology, hormonal profiling and carbohydrate turnover in carnation stem cuttings. Physiol. Plant..

[32] Villacorta-Martín C (2015). Gene expression profiling during adventitious root formation in carnation stem cuttings. BMC Genom..

[33] Kelley DR, Estelle M (2012). Ubiquitin-mediated control of plant hormone signaling. Plant Physiol..

[34] Calderon-Villalobos LI, Tan X, Zheng N, Estelle M (2010). Auxin perception—Structural insights. Cold Spring Harb. Perspect. Biol..

[35] Vierstra RD (2003). The ubiquitin/26S proteasome pathway, the complex last chapter in the life of many plant proteins. Trends Plant Sci..

[36] Salehin M, Bagchi R, Estelle M (2015). SCFTIR1/AFB-based auxin perception: Mechanism and role in plant growth and development. Plant Cell.

[37] Villalobos LIAC (2012). A combinatorial TIR1/AFB–Aux/IAA co-receptor system for differential sensing of auxin. Nat. Chem. Biol..

[38] Tan X (2007). Mechanism of auxin perception by the TIR1 ubiquitin ligase. Nature.

[39] Walsh TA (2006). Mutations in an auxin receptor homolog AFB5 and in SGT1b confer resistance to synthetic picolinate auxins and not to 2, 4-dichlorophenoxyacetic acid or indole-3-acetic acid in Arabidopsis. Plant Physiol..

[40] Dreher KA, Brown J, Saw RE, Callis J (2006). The Arabidopsis Aux/IAA protein family has diversified in degradation and auxin responsiveness. Plant Cell.

[41] Tiwari SB, Hagen G, Guilfoyle TJ (2004). Aux/IAA proteins contain a potent transcriptional repression domain. Plant Cell.

[42] Weijers D (2005). Developmental specificity of auxin response by pairs of ARF and Aux/IAA transcriptional regulators. EMBO J..

[43] Parry G (2009). Complex regulation of the TIR1/AFB family of auxin receptors. Proc. Natl. Acad. Sci..

[44] Peng Y, Fang T, Zhang Y, Zhang M, Zeng L (2020). Genome-wide identification and expression analysis of auxin response factor (ARF) gene family in Longan (*Dimocarpus longan* L.). Plants.

[45] Li S-B (2015). Genome-wide identification, isolation and expression analysis of auxin response factor (ARF) gene family in sweet orange (*Citrus sinensis*). Front. Plant Sci..

[46] Piya S, Shrestha SK, Binder B, Stewart CN, Hewezi T (2014). Protein–protein interaction and gene co-expression maps of ARFs and Aux/IAAs in Arabidopsis. Front. Plant Sci..

[47] Finet C, Berne-Dedieu A, Scutt CP, Marlétaz F (2013). Evolution of the ARF gene family in land plants: Old domains, new tricks. Mol. Biol. Evol..

[48] Li S-B, Xie Z-Z, Hu C-G, Zhang J-Z (2016). A review of auxin response factors (ARFs) in plants. Front. Plant Sci..

[49] Bailey TL (2009). MEME SUITE: Tools for motif discovery and searching. Nucleic Acids Res..

[50] Xie R (2015). The ARF, AUX/IAA and GH3 gene families in citrus: Genome-wide identification and expression analysis during fruitlet drop from abscission zone A. Mol. Genet. Genom..

[51] Jiao Y (2011). Ancestral polyploidy in seed plants and angiosperms. Nature.

[52] Overvoorde P, Fukaki H, Beeckman T (2010). Auxin control of root development. Cold Spring Harb. Perspect. Biol..

[53] Ha M, Kim VN (2014). Regulation of microRNA biogenesis. Nat. Rev. Mol. Cell Biol..

[54] Llave C, Xie Z, Kasschau KD, Carrington JC (2002). Cleavage of scarecrow-like mRNA targets directed by a class of Arabidopsis miRNA. Science.

[55] Song L, Axtell MJ, Fedoroff NV (2010). RNA secondary structural determinants of miRNA precursor processing in Arabidopsis. Curr. Biol..

[56] Wang L, Mai Y-X, Zhang Y-C, Luo Q, Yang H-Q (2010). MicroRNA171c-targeted SCL6-II, SCL6-III, and SCL6-IV genes regulate shoot branching in Arabidopsis. Mol. Plant.

[57] Yu N, Niu QW, Ng KH, Chua NH (2015). The role of miR156/SPL s modules in Arabidopsis lateral root development. Plant J..

[58] Xu X (2017). High miR156 expression is required for auxin-induced adventitious root formation via MxSPL26 independent of PINs and ARFs in *Malus xiaojinensis*. Front. Plant Sci..

[59] Bustos-Sanmamed P (2013). Overexpression of miR160 affects root growth and nitrogen-fixing nodule number in *Medicago truncatula*. Funct. Plant Biol..

[60] Lin Y (2015). Endogenous target mimics down-regulate miR160 mediation of ARF10,-16, and-17 cleavage during somatic embryogenesis in *Dimocarpus longan* Lour. Front. Plant Sci..

[61] Liu S, Wu L, Qi H, Xu M (2019). LncRNA/circRNA–miRNA–mRNA networks regulate the development of root and shoot meristems of Populus. Ind. Crops Prod..

[62] Zhang B, Pan X, Cobb GP, Anderson TA (2006). Plant microRNA: A small regulatory molecule with big impact. Dev. Biol..

[63] Park J-E (2007). GH3-mediated auxin homeostasis links growth regulation with stress adaptation response in Arabidopsis. J. Biol. Chem..

[64] Gutierrez L (2012). Auxin controls Arabidopsis adventitious root initiation by regulating jasmonic acid homeostasis. Plant Cell.

[65] Leyser O (2018). Auxin signaling. Plant Physiol..

[66] Iwakawa H-O, Tomari Y (2015). The functions of microRNAs: mRNA decay and translational repression. Trends Cell Biol..

[67] Grabherr MG (2011). Full-length transcriptome assembly from RNA-Seq data without a reference genome. Nat. Biotechnol..

[68] Pertea G (2003). TIGR Gene Indices clustering tools (TGICL): A software system for fast clustering of large EST datasets. Bioinformatics.

[69] Simão FA, Waterhouse RM, Ioannidis P, Kriventseva EV, Zdobnov EM (2015). BUSCO: Assessing genome assembly and annotation completeness with single-copy orthologs. Bioinformatics.

[70] Trapnell C (2012). Differential gene and transcript expression analysis of RNA-seq experiments with TopHat and Cufflinks. Nat. Protoc..

[71] Wang L, Feng Z, Wang X, Wang X, Zhang X (2010). DEGseq: An R package for identifying differentially expressed genes from RNA-seq data. Bioinformatics.

[72] Evers M, Huttner M, Dueck A, Meister G, Engelmann JC (2015). miRA: Adaptable novel miRNA identification in plants using small RNA sequencing data. BMC Bioinform..

[73] Hoen PA (2008). Deep sequencing-based expression analysis shows major advances in robustness, resolution and inter-lab portability over five microarray platforms. Nucleic Acids Res..

[74] Anders, S. & Huber, W. Differential expression analysis for sequence count data. *Nat. Prec*. 1–1 (2010). 10.1186/gb-2010-11-10-r106 PMC3218662 20979621

[75] Bo X, Wang S (2005). TargetFinder: A software for antisense oligonucleotide target site selection based on MAST and secondary structures of target mRNA. Bioinformatics.

[76] Addo-Quaye C, Miller W, Axtell MJ (2009). CleaveLand: A pipeline for using degradome data to find cleaved small RNA targets. Bioinformatics.

[77] Meyers BC (2008). Criteria for annotation of plant MicroRNAs. Plant Cell.

[78] Zuker M, Stiegler P (1981). Optimal computer folding of large RNA sequences using thermodynamics and auxiliary information. Nucleic Acids Res..

[79] Kanehisa M, Goto S (2000). KEGG: Kyoto encyclopedia of genes and genomes. Nucleic Acids Res..

[80] Kanehisa M (2019). Toward understanding the origin and evolution of cellular organisms. Protein Sci..

[81] Kanehisa M (2021). KEGG: Integrating viruses and cellular organisms. Nucleic Acids Res..

[82] Chen C (2020). TBtools: An integrative toolkit developed for interactive analyses of big biological data. Mol. Plant.

[83] Peng, Y., Fang, T., Zhang, Y., Zhang, M. & Zeng, L. Genome-wide identification and expression analysis of auxin response factor (ARF) gene family in Longan (*Dimocarpus longan* L.). *Plants (Basel)* **9** (2020). 10.3390/plants9020221 PMC7076634 32046357

[84] El-Gebali S (2019). The Pfam protein families database in 2019. Nucleic Acids Res..

[85] Kumar S, Stecher G, Tamura K (2016). MEGA7: Molecular evolutionary genetics analysis version 7.0 for bigger datasets. Mol. Biol. Evolut..

[86] Nei M, Kumar S (2000). Molecular Evolution and Phylogenetics.

[87] Bailey TL, Elkan C (1995). The value of prior knowledge in discovering motifs with MEME. ISMB..

[88] Chen, C. *et al.* Tbtools—An integrative toolkit developed for interactive analyses of big biological data. *bioRxiv*. 289660 (2020). 10.1016/j.molp.2020.06.009 32585190

[89] Lin YL, Lai ZX (2013). Evaluation of suitable reference genes for normalization of microRNA expression by real-time reverse transcription PCR analysis during longan somatic embryogenesis. Plant Physiol. Biochem..

[90] Zhu L (2020). Reference gene selection for quantitative real-time PCR analyses of *Acer palmatum* under abiotic stress. Phyton.

[91] Livak KJ, Schmittgen TD (2001). Analysis of relative gene expression data using real-time quantitative PCR and the 2^−ΔΔCT^ method. Methods.

[92] Lichocka M, Schmelzer E (2014). Subcellular localization experiments and FRET-FLIM measurements in plants. Bio-Protoc..

[93] Sorin C (2005). Auxin and light control of adventitious rooting in Arabidopsis require ARGONAUTE1. Plant Cell.

[94] Collins TJ (2007). ImageJ for microscopy. Biotechniques.

