# Supplementary material for: Key regulatory pathways, microRNAs, and target genes participate in adventitious root formation of Acer rubrum L
Source: Sci Rep. 2022 Jul 14;12:12057. doi: 10.1038/s41598-022-16255-7 (PMC9283533; doi:10.1038/s41598-022-16255-7)
Supplement: Supplementary file 1 — Supplementary Information. [file 41598_2022_16255_MOESM1_ESM.docx]

Supplementary Material

## Supplementary Tables

**Table S1.** Identification of known miRNAs.

| **miRNA id** | **nt** | **CK count** | **IBA300 count** | **Mature sequences** |
| --- | --- | --- | --- | --- |
| miR156c_1 | 19 | 44 | 22 | TGACAGAAGAGAGGGAGCA |
| miR156f | 22 | 111 | 91 | TTGACAGAAGAGAGAGAGCACA |
| miR159a- 5p | 20 | 345 | 82 | TTTGGATTGAAGGGAGCTCTA |
| miR159a_1 | 20 | 168003 | 203532 | AGCTGCTGAGCTATGGATCCC |
| miR160 | 21 | 28022 | 136 | TGGCATACAGGGAGCCAGGCA |
| miR160a3p_2 | 21 | 516 | 448 | GCGTATGAGGAGCCATGCATA |
| miR160a-5p | 20 | 12889 | 2661 | TGCCTGGCTCCCTGTATGCCA |
| miR160a5p_1 | 20 | 138 | 7 | GCCTGGCTCCCTGTATGCCAT |
| miR160b_1 | 19 | 1407 | 1199 | TGCCTGGCTCCCTGTATGCC |
| miR160h | 22 | 5 | 4 | TGCCTGGCTCCCTGTATGCCATT |
| miR162a-5p | 22 | 118 | 110 | TGGAGGCAGCGGTTCATCGATC |
| miR162a5p_1 | 21 | 97 | 31 | GGAGGCAGCGGTTCATCGATC |
| miR166 | 22 | 1527 | 928 | TCGGACCAGGCTTCATTCCCCC |
| miR166a-3p | 21 | 331208 | 319279 | TCGGACCAGGCTTCATTCCCC |
| miR166a5p_1 | 21 | 184 | 229 | GGAATGTTGTCTGGTTCAAGG |
| miR166d5p_2 | 21 | 7321 | 3740 | GGAATGTTGTCTGGCTCGAGG |
| miR166e | 19 | 2618 | 1114 | GGACCAGGCTTCATTCCCC |
| miR166e-3p | 21 | 2374 | 3616 | CTCGGACCAGGCTTCATTCCC |
| miR166h-3p | 20 | 3019 | 4114 | TCGGACCAGGCTTCATTCCC |
| miR166m_2 | 20 | 2751 | 6874 | CGGACCAGGCTTCATTCCCC |
| miR167a_2 | 22 | 12597 | 23314 | TGAAGCTGCCAGCATGATCTGA |
| miR167d-5p | 20 | 5834 | 11457 | TGAAGCTGCCAGCATGATCT |
| miR167d_1 | 21 | 7940 | 10458 | TGAAGCTGCCAGCATGATCTG |
| miR171a-3p | 21 | 5871 | 11564 | TGATTGAGCCGCGCCAATATC |
| miR171a3p_1 | 20 | 29 | 39 | TGATTGAGCCGCGCCAATAT |
| miR171d_1 | 20 | 2817 | 891 | TTGAGCCGCGCCAATATCAC |
| miR171f_3 | 21 | 2339 | 1622 | TTGAGCCGCGCCAATATCACT |
| miR172a_2 | 20 | 20 | 47 | AGAATCTTGATGATGCTGCA |
| miR172a_3 | 21 | 28 | 30 | AGAATCTTGATGATGCTGCAT |
| miR172b5p_2 | 20 | 6 | 12 | GTAGCATCATCAAGATTCAC |
| miR172c-3p | 19 | 3 | 5 | AGAATCTTGATGATGCTGC |
| miR172c-5p | 21 | 45 | 67 | GTAGCATCATCAAGATTCACA |
| miR172e3p_1 | 20 | 22 | 59 | GAATCTTGATGATGCTGCAT |
| miR390a-5p | 22 | 8733 | 5935 | AAGCTCAGGAGGGATAGCGCC |
| miR390e | 21 | 416 | 712 | AGCTCAGGAGGGATAGCGCC |
| miR396-3p_1 | 22 | 1 | 1 | AAGCTCAAGAAAGCTGTGGGA |
| miR396a3p_1 | 20 | 2738 | 1130 | CACAGCTTTCTTGAACTTTCT |
| miR396a3p_4 | 21 | 2173 | 1696 | GTTCAATAAAGCTGTGGGAAG |
| miR396a_1 | 20 | 5 | 3 | GTTCAATAAAGCTGTGGGAA |
| miR396b | 19 | 11423 | 6604 | TTCCACAGCTTTCTTGAACT |
| **miRNA id** | **nt** | **CK count** | **IBA300 count** | **Mature sequences** |
| miR396b3p_2 | 21 | 474 | 336 | GCTCAAGAAAGCTGTGGGAGA |
| miR396b-5p | 19 | 10020 | 15876 | TTCCACAGCTTTCTTGAACTT |
| miR396e3p_3 | 20 | 264 | 323 | CTCAAGAAAGCTGTGGGAGA |
| miR482d3p_2 | 22 | 9610 | 18291 | TCCCTACTCCACCCATGCCATA |
| miR5021 | 20 | 0 | 1 | TGAGAAGAAGAAGAAGAAAA |
| miR5532 | 21 | 0 | 1 | ATGGAATATATGACAAAGGTGG |
| miR8175 | 20 | 415 | 438 | GATCCCCGGCAACGGCGCCA |
| miR827_2 | 20 | 94 | 15 | TTAGATGACCATCAACGAACA |

**Table S2.** Number of miRNA-mRNA pairs identified by the degradome libraries

| **SmallRNA** | **SmallRNA_seq** | **Transcript** |
| --- | --- | --- |
| miR172a_2 | AGAATCTTGATGATGCTGCA | Unigene10671_All |
| miR160a_5p | TGCCTGGCTCCCTGTATGCCA | CL3897.Contig1_All |
| miR5021 | TGAGAAGAAGAAGAAGAAAA | CL11694.Contig1_All |
| miR172c_3p | AGAATCTTGATGATGCTGC | CL321.Contig8_All |
| miR172a_3 | AGAATCTTGATGATGCTGCAT | Unigene8113_All |
| miR160h | TGCCTGGCTCCCTGTATGCCATT | CL3897.Contig1_All |
| miR171d_1 | TTGAGCCGCGCCAATATCAC | Unigene23148_All |
| miR5021 | TGAGAAGAAGAAGAAGAAAA | Unigene15695_All |
| miR5021 | TGAGAAGAAGAAGAAGAAAA | CL10877.Contig2_All |
| miR172a_2 | AGAATCTTGATGATGCTGCA | CL321.Contig7_All |
| miR172a_2 | AGAATCTTGATGATGCTGCA | Unigene8113_All |
| miR396b | TTCCACAGCTTTCTTGAACT | Unigene25911_All |
| novel_mir41 | AGATGGTTCGATTAGTCTTTCGCC | CL7474.Contig1_All |
| miR171f_3 | TTGAGCCGCGCCAATATCACT | CL2444.Contig4_All |
| miR172a_2 | AGAATCTTGATGATGCTGCA | CL321.Contig9_All |
| miR166h_3p | TCGGACCAGGCTTCATTCCC | CL5602.Contig1_All |
| miR166a_3p | TCGGACCAGGCTTCATTCCCC | CL5602.Contig1_All |
| miR172a_2 | AGAATCTTGATGATGCTGCA | CL321.Contig8_All |
| miR171d_1 | TTGAGCCGCGCCAATATCAC | CL2444.Contig4_All |
| miR160a_5p | TGCCTGGCTCCCTGTATGCCA | Unigene5190_All |
| miR172c_3p | AGAATCTTGATGATGCTGC | Unigene8113_All |
| miR160h | TGCCTGGCTCCCTGTATGCCATT | Unigene5190_All |
| miR160b_1 | TGCCTGGCTCCCTGTATGCC | Unigene5190_All |
| miR171f_3 | TTGAGCCGCGCCAATATCACT | Unigene23148_All |
| miR172c_3p | AGAATCTTGATGATGCTGC | CL321.Contig9_All |
| miR172a_3 | AGAATCTTGATGATGCTGCAT | Unigene10671_All |
| miR160b_1 | TGCCTGGCTCCCTGTATGCC | CL3897.Contig1_All |
| miR171a_3p_1 | TGATTGAGCCGCGCCAATAT | Unigene10315_All |
| miR172a_3 | AGAATCTTGATGATGCTGCAT | CL321.Contig9_All |
| miR172a_3 | AGAATCTTGATGATGCTGCAT | CL321.Contig8_All |
| miR172a_3 | AGAATCTTGATGATGCTGCAT | CL321.Contig7_All |
| miR166 | TCGGACCAGGCTTCATTCCCCC | CL5602.Contig1_All |
| miR172c_3p | AGAATCTTGATGATGCTGC | Unigene10671_All |
| miR171a_3p | TGATTGAGCCGCGCCAATATC | Unigene10315_All |
| miR172c_3p | AGAATCTTGATGATGCTGC | CL321.Contig7_All |
| miR156f | TTGACAGAAGAGAGAGAGCACA | CL2023.Contig4_All |
| miR156f | TTGACAGAAGAGAGAGAGCACA | CL2023.Contig5_All |
| miR156f | TTGACAGAAGAGAGAGAGCACA | CL2023.Contig2_All |
| miR156f | TTGACAGAAGAGAGAGAGCACA | CL2023.Contig1_All |
| miR156f | TTGACAGAAGAGAGAGAGCACA | CL2023.Contig3_All |
| miR171a_3p | TGATTGAGCCGCGCCAATATC | Unigene23148_All |
| miR171a_3p_1 | TGATTGAGCCGCGCCAATAT | Unigene23148_All |
| miR5021 | TGAGAAGAAGAAGAAGAAAA | CL14529.Contig1_All |
| miR5021 | TGAGAAGAAGAAGAAGAAAA | Unigene868_All |
| novel_mir21 | AATGGTTCACATTCTTCGTCCTAATATATT | Unigene18173_All |
| novel_mir21 | AATGGTTCACATTCTTCGTCCTAATATATT | CL9773.Contig2_All |
| miR172a_2 | AGAATCTTGATGATGCTGCA | CL4031.Contig4_All |
| miR172c_3p | AGAATCTTGATGATGCTGC | CL4031.Contig4_All |
| miR172c_3p | AGAATCTTGATGATGCTGC | CL4031.Contig3_All |
| **SmallRNA** | **SmallRNA_seq** | **Transcript** |
| miR172a_2 | AGAATCTTGATGATGCTGCA | CL4031.Contig3_All |
| miR396b_5p | TTCCACAGCTTTCTTGAACTT | Unigene16323_All |
| miR396b | TTCCACAGCTTTCTTGAACT | Unigene16323_All |
| miR390e | AGCTCAGGAGGGATAGCGCC | Unigene18779_All |
| miR171a_3p_1 | TGATTGAGCCGCGCCAATAT | CL2444.Contig4_All |
| miR171a_3p | TGATTGAGCCGCGCCAATATC | CL2444.Contig4_All |
| miR156c_1 | TGACAGAAGAGAGGGAGCA | Unigene23422_All |
| miR5021 | TGAGAAGAAGAAGAAGAAAA | CL8284.Contig1_All |
| miR5021 | TGAGAAGAAGAAGAAGAAAA | CL10745.Contig1_All |
| miR172c_5p | GTAGCATCATCAAGATTCACA | Unigene5271_All |
| miR5021 | TGAGAAGAAGAAGAAGAAAA | CL10745.Contig2_All |
| miR172b_5p_2 | GTAGCATCATCAAGATTCAC | Unigene5271_All |
| miR396b | TTCCACAGCTTTCTTGAACT | CL104.Contig3_All |
| novel_mir78 | GAAGAATGTGGACCACTAG | CL464.Contig2_All |
| miR172a_3 | AGAATCTTGATGATGCTGCAT | CL11586.Contig1_All |
| miR172c_3p | AGAATCTTGATGATGCTGC | CL11586.Contig3_All |
| miR172a_2 | AGAATCTTGATGATGCTGCA | CL11586.Contig3_All |
| miR172a_2 | AGAATCTTGATGATGCTGCA | CL11586.Contig1_All |
| miR172c_3p | AGAATCTTGATGATGCTGC | CL11586.Contig1_All |
| miR172a_3 | AGAATCTTGATGATGCTGCAT | CL11586.Contig3_All |
| novel_mir82 | TAGTGGTTCACATTTTTCGTCCTAATA | CL9773.Contig2_All |
| miR5021 | TGAGAAGAAGAAGAAGAAAA | CL913.Contig2_All |
| novel_mir23 | ATGTGGGATGTGGGATAAGGGATAAAAAGT | CL501.Contig1_All |
| novel_mir82 | TAGTGGTTCACATTTTTCGTCCTAATA | Unigene18173_All |
| novel_mir23 | ATGTGGGATGTGGGATAAGGGATAAAAAGT | CL501.Contig2_All |
| miR5021 | TGAGAAGAAGAAGAAGAAAA | CL913.Contig1_All |
| miR172b_5p_2 | GTAGCATCATCAAGATTCAC | Unigene10671_All |
| miR172c_5p | GTAGCATCATCAAGATTCACA | Unigene10671_All |
| miR396b | TTCCACAGCTTTCTTGAACT | Unigene51067_All |
| miR5021 | TGAGAAGAAGAAGAAGAAAA | Unigene3346_All |
| miR396a_1 | CACAGCTTTCTTGAACTTTCT | Unigene170_All |
| miR5021 | TGAGAAGAAGAAGAAGAAAA | Unigene13254_All |
| miR390a_5p | AAGCTCAGGAGGGATAGCGCC | Unigene13128_All |
| novel_mir45 | TGGACCACTAGATTTACATACTTTGACTCA | Unigene53632_All |
| miR172e_3p_1 | GAATCTTGATGATGCTGCAT | CL321.Contig7_All |
| miR172e_3p_1 | GAATCTTGATGATGCTGCAT | CL321.Contig8_All |
| miR172e_3p_1 | GAATCTTGATGATGCTGCAT | CL321.Contig9_All |
| miR5021 | TGAGAAGAAGAAGAAGAAAA | Unigene8209_All |
| novel_mir9 | TCCACATTCTTCGTCATAATATATCTT | Unigene35117_All |
| miR5021 | TGAGAAGAAGAAGAAGAAAA | CL11071.Contig1_All |
| miR172e_3p_1 | GAATCTTGATGATGCTGCAT | Unigene8113_All |
| miR5021 | TGAGAAGAAGAAGAAGAAAA | CL11682.Contig2_All |
| miR5021 | TGAGAAGAAGAAGAAGAAAA | Unigene809_All |
| miR396a_1 | CACAGCTTTCTTGAACTTTCT | Unigene3038_All |
| novel_mir78 | GAAGAATGTGGACCACTAG | CL2847.Contig2_All |
| novel_mir78 | GAAGAATGTGGACCACTAG | CL3860.Contig2_All |
| novel_mir78 | GAAGAATGTGGACCACTAG | Unigene53632_All |
| miR166e_3p | CTCGGACCAGGCTTCATTCCC | CL5602.Contig1_All |
| novel_mir20 | CGGTAAGGACCACCATGAAATCATATCGA | CL32.Contig12_All |
| miR396b | TTCCACAGCTTTCTTGAACT | Unigene23737_All |
| **SmallRNA** | **SmallRNA_seq** | **Transcript** |
| miR5021 | TGAGAAGAAGAAGAAGAAAA | CL12966.Contig1_All |
| miR5021 | TGAGAAGAAGAAGAAGAAAA | CL3248.Contig5_All |
| novel_mir39 | GATCTGGAAGAGAGTCGACCGTTGGAGAAG | Unigene15343_All |
| novel_mir68 | CCACATTGTTCGTCCTGATATATTTTATA | Unigene18173_All |
| novel_mir68 | CCACATTGTTCGTCCTGATATATTTTATA | CL9773.Contig2_All |
| miR156f | TTGACAGAAGAGAGAGAGCACA | CL4258.Contig2_All |
| miR5021 | TGAGAAGAAGAAGAAGAAAA | CL11576.Contig2_All |
| miR156f | TTGACAGAAGAGAGAGAGCACA | CL4258.Contig1_All |
| miR156f | TTGACAGAAGAGAGAGAGCACA | CL4258.Contig3_All |
| miR396b_5p | TTCCACAGCTTTCTTGAACTT | Unigene11965_All |
| miR396b_5p | TTCCACAGCTTTCTTGAACTT | Unigene8418_All |
| novel_mir45 | TGGACCACTAGATTTACATACTTTGACTCA | Unigene20588_All |
| miR396b_5p | TTCCACAGCTTTCTTGAACTT | CL13632.Contig1_All |
| miR396b | TTCCACAGCTTTCTTGAACT | Unigene8418_All |
| novel_mir39 | GATCTGGAAGAGAGTCGACCGTTGGAGAAG | Unigene15344_All |
| novel_mir46 | AATTCTATCGGGATATTGAATGTGA | CL2566.Contig3_All |
| miR396b | TTCCACAGCTTTCTTGAACT | Unigene11965_All |
| miR172c_3p | AGAATCTTGATGATGCTGC | Unigene3299_All |
| miR5021 | TGAGAAGAAGAAGAAGAAAA | CL6955.Contig2_All |
| miR396b | TTCCACAGCTTTCTTGAACT | CL13632.Contig1_All |
| miR156c_1 | TGACAGAAGAGAGGGAGCA | CL12778.Contig2_All |
| miR5021 | TGAGAAGAAGAAGAAGAAAA | Unigene35466_All |
| miR5021 | TGAGAAGAAGAAGAAGAAAA | Unigene13046_All |
| novel_mir78 | GAAGAATGTGGACCACTAG | CL14128.Contig3_All |
| miR5021 | TGAGAAGAAGAAGAAGAAAA | CL11682.Contig2_All |
| miR396b | TTCCACAGCTTTCTTGAACT | Unigene20721_All |
| novel_mir78 | GAAGAATGTGGACCACTAG | CL14128.Contig4_All |
| miR5021 | TGAGAAGAAGAAGAAGAAAA | Unigene7463_All |
| miR5021 | TGAGAAGAAGAAGAAGAAAA | Unigene20660_All |
| novel_mir83 | TGTCACAAAGTTTGCCAATA | CL12673.Contig1_All |
| miR5021 | TGAGAAGAAGAAGAAGAAAA | Unigene8237_All |
| miR172c_3p | AGAATCTTGATGATGCTGC | Unigene5441_All |
| novel_mir78 | GAAGAATGTGGACCACTAG | CL14128.Contig2_All |
| miR5021 | TGAGAAGAAGAAGAAGAAAA | Unigene10493_All |
| miR396b | TTCCACAGCTTTCTTGAACT | CL13612.Contig2_All |
| novel_mir80 | AAAATATATCAGGACGAAGAATGT | CL2847.Contig2_All |
| miR172c_3p | AGAATCTTGATGATGCTGC | CL1676.Contig3_All |
| miR172a_2 | AGAATCTTGATGATGCTGCA | CL1676.Contig3_All |
| miR172e_3p_1 | GAATCTTGATGATGCTGCAT | CL930.Contig2_All |
| miR5021 | TGAGAAGAAGAAGAAGAAAA | CL4763.Contig1_All |
| miR5021 | TGAGAAGAAGAAGAAGAAAA | CL11576.Contig3_All |
| miR5021 | TGAGAAGAAGAAGAAGAAAA | Unigene58291_All |
| novel_mir83 | TGTCACAAAGTTTGCCAATA | CL1072.Contig8_All |
| miR156c_1 | TGACAGAAGAGAGGGAGCA | CL219.Contig1_All |
| miR396b_5p | TTCCACAGCTTTCTTGAACTT | Unigene11378_All |
| miR396b | TTCCACAGCTTTCTTGAACT | Unigene11378_All |
| miR160a_5p_1 | GCCTGGCTCCCTGTATGCCAT | CL3897.Contig1_All |
| miR167d_1 | TGAAGCTGCCAGCATGATCT | Unigene23399_All |
| miR396b_5p | TTCCACAGCTTTCTTGAACTT | Unigene25263_All |
| miR396b | TTCCACAGCTTTCTTGAACT | Unigene25263_All |
| **SmallRNA** | **SmallRNA_seq** | **Transcript** |
| miR172a_2 | AGAATCTTGATGATGCTGCA | CL1676.Contig2_All |
| miR172c_3p | AGAATCTTGATGATGCTGC | CL1676.Contig1_All |
| miR172c_3p | AGAATCTTGATGATGCTGC | CL1676.Contig2_All |
| miR172a_2 | AGAATCTTGATGATGCTGCA | CL1676.Contig1_All |
| miR166e | GGACCAGGCTTCATTCCCC | CL8627.Contig2_All |
| miR5021 | TGAGAAGAAGAAGAAGAAAA | Unigene25143_All |
| miR5021 | TGAGAAGAAGAAGAAGAAAA | CL12892.Contig1_All |
| miR396b_3p_2 | GCTCAAGAAAGCTGTGGGAGA | CL4192.Contig3_All |
| miR156c_1 | TGACAGAAGAGAGGGAGCA | CL11698.Contig2_All |
| miR172c_3p | AGAATCTTGATGATGCTGC | Unigene33332_All |
| miR172c_3p | AGAATCTTGATGATGCTGC | CL8944.Contig2_All |
| novel_mir81 | CACTCGTTTTACATACTTTGACTC | CL9773.Contig2_All |
| miR5021 | TGAGAAGAAGAAGAAGAAAA | Unigene18296_All |
| miR156f | TTGACAGAAGAGAGAGAGCACA | CL219.Contig1_All |
| miR172b_5p_2 | GTAGCATCATCAAGATTCAC | Unigene47979_All |
| miR172c_5p | GTAGCATCATCAAGATTCACA | Unigene47979_All |
| novel_mir47 | CCTGAAGTCATTGTAGTAT | CL2930.Contig1_All |
| novel_mir26 | CATTTCGTTGTGAGAAAGGT | Unigene41272_All |
| miR5021 | TGAGAAGAAGAAGAAGAAAA | CL5225.Contig1_All |
| miR5021 | TGAGAAGAAGAAGAAGAAAA | Unigene5455_All |
| novel_mir26 | CATTTCGTTGTGAGAAAGGT | CL13384.Contig1_All |
| miR166h_3p | TCGGACCAGGCTTCATTCCC | CL5710.Contig1_All |
| novel_mir77 | GTCCTAACTTTGTTATATCAGTTCCTTGAA | Unigene473_All |
| novel_mir69 | AACTTCTCCATAATCTATGGTCCACATTCT | Unigene81133_All |
| miR166h_3p | TCGGACCAGGCTTCATTCCC | CL5710.Contig2_All |
| miR5021 | TGAGAAGAAGAAGAAGAAAA | Unigene9862_All |
| miR5021 | TGAGAAGAAGAAGAAGAAAA | CL3119.Contig2_All |
| miR5021 | TGAGAAGAAGAAGAAGAAAA | Unigene21193_All |
| miR156f | TTGACAGAAGAGAGAGAGCACA | Unigene2962_All |
| novel_mir47 | CCTGAAGTCATTGTAGTAT | CL2930.Contig2_All |
| novel_mir87 | GGATATTGAATGTGAACCACTCATTTATA | CL2566.Contig3_All |
| novel_mir3 | ACGAAAAATGTGGACCACTAGATTTACAT | Unigene20588_All |
| miR396b | TTCCACAGCTTTCTTGAACT | Unigene37961_All |
| miR396b_5p | TTCCACAGCTTTCTTGAACTT | Unigene37961_All |
| miR5021 | TGAGAAGAAGAAGAAGAAAA | CL9139.Contig2_All |
| novel_mir27 | TTTGGGCCCAATTGAATGAAATGTT | CL7116.Contig2_All |
| miR172e_3p_1 | GAATCTTGATGATGCTGCAT | CL11586.Contig3_All |
| miR5021 | TGAGAAGAAGAAGAAGAAAA | CL840.Contig2_All |
| novel_mir80 | AAAATATATCAGGACGAAGAATGT | Unigene85839_All |
| novel_mir27 | TTTGGGCCCAATTGAATGAAATGTT | CL7116.Contig1_All |
| novel_mir80 | AAAATATATCAGGACGAAGAATGT | CL6435.Contig1_All |
| miR5021 | TGAGAAGAAGAAGAAGAAAA | CL840.Contig3_All |
| miR5021 | TGAGAAGAAGAAGAAGAAAA | Unigene23120_All |
| miR5021 | TGAGAAGAAGAAGAAGAAAA | CL840.Contig1_All |
| miR5021 | TGAGAAGAAGAAGAAGAAAA | CL840.Contig4_All |
| miR172e_3p_1 | GAATCTTGATGATGCTGCAT | CL11586.Contig1_All |
| novel_mir80 | AAAATATATCAGGACGAAGAATGT | CL9773.Contig2_All |
| novel_mir80 | AAAATATATCAGGACGAAGAATGT | Unigene35034_All |
| miR396e_3p_3 | CTCAAGAAAGCTGTGGGAGA | CL3152.Contig8_All |
| miR396e_3p_3 | CTCAAGAAAGCTGTGGGAGA | CL3152.Contig7_All |
| **SmallRNA** | **SmallRNA_seq** | **Transcript** |
| miR396e_3p_3 | CTCAAGAAAGCTGTGGGAGA | CL3152.Contig6_All |
| miR396e_3p_3 | CTCAAGAAAGCTGTGGGAGA | Unigene12687_All |
| miR396e_3p_3 | CTCAAGAAAGCTGTGGGAGA | Unigene12667_All |
| miR5021 | TGAGAAGAAGAAGAAGAAAA | Unigene49561_All |
| miR172c_5p | GTAGCATCATCAAGATTCACA | CL2759.Contig1_All |
| miR172b_5p_2 | GTAGCATCATCAAGATTCAC | CL2759.Contig1_All |
| miR172c_3p | AGAATCTTGATGATGCTGC | CL2295.Contig2_All |
| miR5021 | TGAGAAGAAGAAGAAGAAAA | CL7711.Contig1_All |
| miR5021 | TGAGAAGAAGAAGAAGAAAA | CL4978.Contig1_All |
| miR5021 | TGAGAAGAAGAAGAAGAAAA | CL3044.Contig3_All |
| miR5021 | TGAGAAGAAGAAGAAGAAAA | CL1476.Contig2_All |
| miR5021 | TGAGAAGAAGAAGAAGAAAA | CL13522.Contig5_All |
| novel_mir47 | CCTGAAGTCATTGTAGTAT | CL5972.Contig3_All |
| miR5021 | TGAGAAGAAGAAGAAGAAAA | CL5446.Contig2_All |
| miR5021 | TGAGAAGAAGAAGAAGAAAA | CL13522.Contig1_All |
| novel_mir47 | CCTGAAGTCATTGTAGTAT | CL5972.Contig1_All |
| miR5021 | TGAGAAGAAGAAGAAGAAAA | CL3044.Contig4_All |
| miR5021 | TGAGAAGAAGAAGAAGAAAA | CL1476.Contig3_All |
| miR5021 | TGAGAAGAAGAAGAAGAAAA | CL3044.Contig2_All |
| novel_mir80 | AAAATATATCAGGACGAAGAATGT | CL13238.Contig1_All |
| miR5021 | TGAGAAGAAGAAGAAGAAAA | CL3044.Contig6_All |
| novel_mir21 | AATGGTTCACATTCTTCGTCCTAATATATT | CL3974.Contig2_All |
| miR5021 | TGAGAAGAAGAAGAAGAAAA | CL1072.Contig13_All |
| novel_mir21 | AATGGTTCACATTCTTCGTCCTAATATATT | CL3974.Contig1_All |
| novel_mir3 | ACGAAAAATGTGGACCACTAGATTTACAT | Unigene43866_All |
| miR5021 | TGAGAAGAAGAAGAAGAAAA | CL394.Contig4_All |
| miR5021 | TGAGAAGAAGAAGAAGAAAA | CL394.Contig5_All |
| miR5021 | TGAGAAGAAGAAGAAGAAAA | CL394.Contig3_All |
| miR5021 | TGAGAAGAAGAAGAAGAAAA | Unigene34772_All |
| miR5021 | TGAGAAGAAGAAGAAGAAAA | CL464.Contig1_All |
| miR5021 | TGAGAAGAAGAAGAAGAAAA | CL1075.Contig1_All |
| miR5021 | TGAGAAGAAGAAGAAGAAAA | CL1075.Contig4_All |
| miR5021 | TGAGAAGAAGAAGAAGAAAA | CL1075.Contig3_All |
| novel_mir28 | ATTTATGAGAGTTCAGGGGGTT | Unigene72882_All |
| novel_mir3 | ACGAAAAATGTGGACCACTAGATTTACAT | CL9773.Contig2_All |
| novel_mir50 | ATAATCTATGATCCACATTCTTCGTCCTGA | CL9773.Contig2_All |
| novel_mir80 | AAAATATATCAGGACGAAGAATGT | Unigene52145_All |
| miR5021 | TGAGAAGAAGAAGAAGAAAA | CL394.Contig6_All |
| miR5021 | TGAGAAGAAGAAGAAGAAAA | CL5651.Contig3_All |
| miR5021 | TGAGAAGAAGAAGAAGAAAA | CL394.Contig2_All |
| novel_mir71 | ATCAGTTCCTTGAATGTGAGCCACTCATTT | Unigene83003_All |
| novel_mir50 | ATAATCTATGATCCACATTCTTCGTCCTGA | Unigene20588_All |
| miR5021 | TGAGAAGAAGAAGAAGAAAA | CL5651.Contig2_All |
| miR5021 | TGAGAAGAAGAAGAAGAAAA | CL5651.Contig5_All |
| miR5021 | TGAGAAGAAGAAGAAGAAAA | CL394.Contig1_All |
| miR166e | GGACCAGGCTTCATTCCCC | Unigene8233_All |
| miR171d_1 | TTGAGCCGCGCCAATATCAC | Unigene10315_All |
| miR171f_3 | TTGAGCCGCGCCAATATCACT | Unigene10315_All |
| miR166a_3p | TCGGACCAGGCTTCATTCCCC | CL1805.Contig7_All |
| miR166h_3p | TCGGACCAGGCTTCATTCCC | CL1805.Contig7_All |
| **SmallRNA** | **SmallRNA_seq** | **Transcript** |
| miR166 | TCGGACCAGGCTTCATTCCCCC | CL1805.Contig7_All |
| miR5021 | TGAGAAGAAGAAGAAGAAAA | Unigene18535_All |
| novel_mir21 | AATGGTTCACATTCTTCGTCCTAATATATT | Unigene35117_All |
| miR5021 | TGAGAAGAAGAAGAAGAAAA | Unigene4853_All |
| miR5021 | TGAGAAGAAGAAGAAGAAAA | Unigene71692_All |
| miR5021 | TGAGAAGAAGAAGAAGAAAA | CL6192.Contig1_All |
| miR5021 | TGAGAAGAAGAAGAAGAAAA | CL1648.Contig1_All |
| miR5021 | TGAGAAGAAGAAGAAGAAAA | Unigene14276_All |
| miR5021 | TGAGAAGAAGAAGAAGAAAA | CL14287.Contig1_All |
| novel_mir69 | AACTTCTCCATAATCTATGGTCCACATTCT | CL11495.Contig1_All |
| novel_mir49 | TCAGGACGAAGAATGTGGATCATAGA | Unigene53632_All |
| novel_mir49 | TCAGGACGAAGAATGTGGATCATAGA | CL3860.Contig2_All |
| miR5021 | TGAGAAGAAGAAGAAGAAAA | CL6247.Contig2_All |
| miR156c_1 | TGACAGAAGAGAGGGAGCA | CL4485.Contig4_All |
| miR156c_1 | TGACAGAAGAGAGGGAGCA | CL4485.Contig7_All |
| miR156c_1 | TGACAGAAGAGAGGGAGCA | CL4485.Contig1_All |
| miR172b_5p_2 | GTAGCATCATCAAGATTCAC | CL7206.Contig2_All |
| miR172b_5p_2 | GTAGCATCATCAAGATTCAC | Unigene35145_All |
| miR172b_5p_2 | GTAGCATCATCAAGATTCAC | CL7206.Contig3_All |
| novel_mir69 | AACTTCTCCATAATCTATGGTCCACATTCT | Unigene52396_All |
| novel_mir69 | AACTTCTCCATAATCTATGGTCCACATTCT | CL9773.Contig2_All |
| miR160a_5p_1 | GCCTGGCTCCCTGTATGCCAT | Unigene5190_All |
| novel_mir69 | AACTTCTCCATAATCTATGGTCCACATTCT | Unigene35116_All |
| miR167d_1 | TGAAGCTGCCAGCATGATCT | Unigene236_All |
| miR156f | TTGACAGAAGAGAGAGAGCACA | Unigene23422_All |
| miR172c_3p | AGAATCTTGATGATGCTGC | Unigene13446_All |
| novel_mir69 | AACTTCTCCATAATCTATGGTCCACATTCT | Unigene35118_All |
| miR5021 | TGAGAAGAAGAAGAAGAAAA | CL13087.Contig2_All |
| miR5021 | TGAGAAGAAGAAGAAGAAAA | CL13087.Contig1_All |
| miR5021 | TGAGAAGAAGAAGAAGAAAA | Unigene35366_All |
| miR5021 | TGAGAAGAAGAAGAAGAAAA | CL3732.Contig1_All |
| novel_mir81 | CACTCGTTTTACATACTTTGACTC | Unigene7811_All |
| novel_mir81 | CACTCGTTTTACATACTTTGACTC | Unigene35643_All |
| novel_mir81 | CACTCGTTTTACATACTTTGACTC | Unigene473_All |
| miR396_3p_1 | AAGCTCAAGAAAGCTGTGGGA | Unigene13441_All |
| miR5021 | TGAGAAGAAGAAGAAGAAAA | CL14097.Contig2_All |
| novel_mir71 | ATCAGTTCCTTGAATGTGAGCCACTCATTT | Unigene17792_All |
| novel_mir71 | ATCAGTTCCTTGAATGTGAGCCACTCATTT | CL10610.Contig8_All |
| miR5021 | TGAGAAGAAGAAGAAGAAAA | CL5994.Contig1_All |
| miR5021 | TGAGAAGAAGAAGAAGAAAA | CL5994.Contig3_All |
| miR5021 | TGAGAAGAAGAAGAAGAAAA | CL5994.Contig2_All |
| novel_mir69 | AACTTCTCCATAATCTATGGTCCACATTCT | Unigene20588_All |
| novel_mir71 | ATCAGTTCCTTGAATGTGAGCCACTCATTT | Unigene35643_All |
| miR5021 | TGAGAAGAAGAAGAAGAAAA | CL10170.Contig2_All |
| miR5021 | TGAGAAGAAGAAGAAGAAAA | CL10170.Contig1_All |
| miR172a_3 | AGAATCTTGATGATGCTGCAT | CL930.Contig2_All |
| miR172a_2 | AGAATCTTGATGATGCTGCA | CL930.Contig2_All |
| miR172c_3p | AGAATCTTGATGATGCTGC | CL930.Contig2_All |
| miR172e_3p_1 | GAATCTTGATGATGCTGCAT | CL930.Contig11_All |
| miR172c_3p | AGAATCTTGATGATGCTGC | CL930.Contig11_All |
| **SmallRNA** | **SmallRNA_seq** | **Transcript** |
| miR172a_2 | AGAATCTTGATGATGCTGCA | CL930.Contig11_All |
| miR172a_3 | AGAATCTTGATGATGCTGCAT | CL930.Contig11_All |
| miR5021 | TGAGAAGAAGAAGAAGAAAA | Unigene20593_All |
| miR5021 | TGAGAAGAAGAAGAAGAAAA | CL7886.Contig2_All |
| miR5021 | TGAGAAGAAGAAGAAGAAAA | CL7886.Contig1_All |
| miR5021 | TGAGAAGAAGAAGAAGAAAA | CL7886.Contig3_All |
| novel_mir3 | ACGAAAAATGTGGACCACTAGATTTACAT | CL3860.Contig2_All |
| novel_mir3 | ACGAAAAATGTGGACCACTAGATTTACAT | Unigene53632_All |
| miR156c_1 | TGACAGAAGAGAGGGAGCA | CL2023.Contig5_All |
| miR156c_1 | TGACAGAAGAGAGGGAGCA | CL2023.Contig1_All |
| miR156c_1 | TGACAGAAGAGAGGGAGCA | CL2023.Contig2_All |
| miR156c_1 | TGACAGAAGAGAGGGAGCA | CL2023.Contig4_All |
| miR156c_1 | TGACAGAAGAGAGGGAGCA | CL2023.Contig3_All |

**Table S3. Primers used for qRT-PCR**

| **Genes** | | **Primers** | | **Sequences** |
| --- | --- | --- | --- | --- |
| Ar-miR160 | Forward Primer | | TGGCATACAGGGAGCCAGGCA | |
| Ar-miR159a-5p | Forward Primer | | AGCTGCTGAGCTATGGATCCC | |
| Ar-miR160a-3p_2 | Forward Primer | | GGCGTATGAGGAGCCATGCATA | |
| Ar-miR160a | Forward Primer | | TGCCTGGCTCCCTGTATGCCA | |
| Ar-miR160a-5p | Forward Primer | | GCCTGGCTCCCTGTATGCCAT | |
| Ar-miR160h | Forward Primer | | TGCCTGGCTCCCTGTATGCCATT | |
| Ar-miR390a-5p | Forward Primer | | AAGCTCAGGAGGGATAGCGCC | |
| Ar-miR172e-3p_1 | Forward Primer | | GAATCTTGATGATGCTGCAT | |
| Ar-miR827_2 | Forward Primer | | TTAGATGACCATCAACGAACA | |
| Ar-miR166m_2 | Forward Primer | | CGGACCAGGCTTCATTCCCC | |
| Ar-miR156c_1 | Forward Primer | | TGACAGAAGAGAGGGAGCA | |
|  | Universal Reverse Primer | | CTCAACTGGTGTCGTGGAGTC | |
| U6 | Forward Primer | | ACAGAGAAGATTAGCATGGCC | |
|  | Reverse Primer | | GACCAATTCTCGATTTGTGCG | |
| CL1805.Contig7_All | Forward Primer | | AAGGTGGTGGTTCAATCATA | |
|  | Reverse Primer | | TCTGGCTAAGTGCTCGTA | |
| CL219. Contig1_All | Forward Primer | | TGGAATCATCATCAGCATCT | |
|  | Reverse Primer | | TCTTGACCACGAATTGTAAC | |
| CL3897.Contig1_All | Forward Primer | | GTAGACTTCAGGAACTGCCAGAGA | |
|  | Reverse Primer | | ACCAGGCTCGTTGTTGCTTATTG | |
| Unigene5190_All | Forward Primer | | CCGCCTTCCATCTATCTAC | |
|  | Reverse Primer | | CCAGCAGGAGTGTTGTTAT | |
| CL321.Contig8_All | Forward Primer | | GATGAAGAATCTGACCAAGGA | |
|  | Reverse Primer | | GCTTCTACTTCGCTGTCG | |
| CL2901.Contig4_All | Forward Primer | | TTGCTGTTGGAGGTGATG | |
|  | Reverse Primer | | TGCTGTCCTTCCTTCTGA | |
| *Actin6* | Forward Primer | | GGATTCAGTTTCACCCAC | |
|  | Reverse Primer | | TTGATAGCACGATACGAGA | |
| *AtGH3.2* | Forward Primer | | CCATAATTCCGCTCCACAGT | |
|  | Reverse Primer | | ACGCATTCTCCACTGCTTTT | |
| *AtGH3.3* | Forward Primer | | ACAATTCCGCTCCACAGTTC | |
|  | Reverse Primer | | ACGAGTTCCTTGCTCTCCAA | |
| *AtGH3.4* | Forward Primer | | CGTTGGAGATACGTGTGGTG | |
|  | Reverse Primer | | GCAGTTTCATGATCGGTGTG | |
| *AtGH3.5* | Forward Primer | | GTCTTCGAGGACTGCTGCTT | |
|  | Reverse Primer | | ATGTCCCTGGCTCAACAATC | |
| *AtGH3.6* | Forward Primer | | CCTTGTTCCGTTTGATGCTT | |
|  | Reverse Primer | | CGTGTTACCGTTCAAGCAGA | |
| *AtGH3.9* | Forward Primer | | GCAGGACGAAAGAGAGATCG | |
|  | Reverse Primer | | AACGACCCTCCTTGGCTTAT | |
| *AtGH3.17* | Forward Primer | | ATGACCCACCAAAGCTTGAC | |
|  | Reverse Primer | | AATCAAACGTGCCCAAACTC | |
| *ArARF10* | Forward Primer | | GTAGACTTCAGGAACTGCCAGAGA | |
|  | Reverse Primer | | ACCAGGCTCGTTGTTGCTTATTG | |
| *AtARF10* | Forward Primer | | TGTCGTCTCCGTGAAGTTCCTC | |
|  | Reverse Primer | | CGTTACCGTTACCGTCAGAAGAAG | |
| *AtARF16* | Forward Primer | | GGATTCCGTGGTGCTCAGGTAT | |
|  | Reverse Primer | | TAGAAGCCGCCAAGGAGAGTTAG | |
| **Genes** | | **Primers** | | **Sequences** |
|  |  | |  | |
| *AtARF17* | Forward Primer | | GCACCTGATCCAAGTCCTTC | |
|  | Reverse Primer | | GGTGAATAGCTGGGGAGGAT | |
| *AtActin11* | Forward Primer | | GTTCTTTCCCTCTACGCT | |
|  | Reverse Primer | | CTTACGATTTCACGCTCT | |

| **Table S4.**  Expression data of the differentially expressed gene enrich pathways | | |
| --- | --- | --- |
| **Pathway Name** | **CK** | **IBA300** |
| ko04712//Circadian rhythm - plant | 1.576533 | 1.878826 |
| ko04626//Plant-pathogen interaction | 0.924806 | 1.68726 |
| ko04146//Peroxisome | 0.721806 | 1.63556 |
| ko04145//Phagosome | 0.832248 | 1.676157 |
| ko04144//Endocytosis | 0.897339 | 1.616785 |
| ko04141//Protein processing in endoplasmic reticulum | 0.93024 | 1.728017 |
| ko04136//Autophagy - other | 0.268748 | 1.49338 |
| ko04130//SNARE interactions in vesicular transport | 0.660552 | 2.089436 |
| ko04122//Sulfur relay system | 3.380139 | 3.988311 |
| ko04120//Ubiquitin mediated proteolysis | 0.713735 | 1.835474 |
| ko04075//Plant hormone signal transduction | 1.35701 | 1.880097 |
| ko04071//Phosphatidylinositol signaling system | 0.576289 | 1.726927 |
| ko04016//MAPK signaling pathway - plant | 1.445126 | 1.965945 |
| ko03450//Non-homologous end-joining | 0.733069 | 1.25377 |
| ko03440//Homologous recombination | 0.589123 | 1.400485 |
| ko03430//Mismatch repair | 0.822697 | 1.253174 |
| ko03420//Nucleotide excision repair | 0.546926 | 0.763442 |
| ko03410//Base excision repair | 0.937654 | 1.255207 |
| ko03060//Protein export | 0.513948 | 1.356776 |
| ko03050//Proteasome | 0.641811 | 1.514599 |
| ko03040//Spliceosome | 0.37368 | 1.689137 |
| ko03030//DNA replication | 0.875627 | 1.543164 |
| ko03022//Basal transcription factors | 0.323986 | 1.609888 |
| ko03020//RNA polymerase;ko00230 | 1.34172 | 1.79596 |
| ko03018//RNA degradation | 0.651981 | 1.679237 |
| ko03015//mRNA surveillance pathway | 0.614998 | 1.688096 |
| ko03013//RNA transport | 0.787017 | 1.603741 |
| ko03010//Ribosome | 1.029789 | 1.410388 |
| ko03008//Ribosome biogenesis in eukaryotes | 0.563272 | 1.528712 |
| ko02010//ABC transporters | 0.705889 | 1.416108 |
| ko01230//Biosynthesis of amino acids | 0.84266 | 1.600544 |
| ko01212//Fatty acid metabolism | 1.304566 | 1.792671 |
| ko01210//2-Oxocarboxylic acid metabolism | 0.307066 | 1.518113 |
| ko01200//Carbon metabolism | 1.140248 | 1.773567 |
| ko01110//Biosynthesis of secondary metabolites | 1.453317 | 1.802142 |
| ko01100//Metabolic pathways | 1.16667 | 1.761188 |
| ko01040//Biosynthesis of unsaturated fatty acids;ko01213//Fatty acid metabolism | 0.692603 | 1.544106 |
|  |  |  |
| **Pathway Name** | **CK** | **IBA300** |
| ko00970//Aminoacyl-tRNA biosynthesis | 0.518748 | 1.538332 |
| ko00966//Glucosinolate biosynthesis | 0.627022 | 1.335659 |
| ko00965//Betalain biosynthesis | 0.588901 | 1.552984 |
| ko00960//Tropane, piperidine and pyridine alkaloid biosynthesis | 1.445666 | 1.296673 |
| ko00950//Isoquinoline alkaloid biosynthesis | 0.903564 | 0.541778 |
| ko00945//Stilbenoid, diarylheptanoid and gingerol biosynthesis | 1.980625 | 2.618864 |
| ko00944//Flavone and flavonol biosynthesis | 1.465948 | 2.528804 |
| ko00943//Isoflavonoid biosynthesis | 2.352211 | 2.008853 |
| ko00942//Anthocyanin biosynthesis | 2.020757 | 1.896012 |
| ko00941//Flavonoid biosynthesis | 1.354028 | 2.812499 |
| ko00940//Phenylpropanoid biosynthesis | 1.899148 | 1.91823 |
| ko00920//Sulfur metabolism | 1.03356 | 2.064465 |
| ko00910//Nitrogen metabolism | 2.079246 | 1.292353 |
| ko00909//Sesquiterpenoid and triterpenoid biosynthesis | 1.111216 | 1.159815 |
| ko00908//Zeatin biosynthesis | 1.80879 | 2.080867 |
| ko00906//Carotenoid biosynthesis | 1.004958 | 1.406154 |
| ko00905//Brassinosteroid biosynthesis | 0.97753 | 2.449144 |
| ko00904//Diterpenoid biosynthesis | 1.446593 | 1.339651 |
| ko00902//Monoterpenoid biosynthesis | 0.468583 | 1.245831 |
| ko00901//Indole alkaloid biosynthesis | 1.560026 | 1.573657 |
| ko00900//Terpenoid backbone biosynthesis | 0.851185 | 1.903329 |
| ko00860//Porphyrin and chlorophyll metabolism | 0.701853 | 2.01118 |
| ko00790//Folate biosynthesis | 0.52904 | 1.688034 |
| ko00785//Lipoic acid metabolism | 0.213803 | -0.113 |
| ko00780//Biotin metabolism | 0.069098 | 1.621385 |
| ko00770//Pantothenate and CoA biosynthesis | -0.12515 | 1.914686 |
| ko00760//Nicotinate and nicotinamide metabolism | 0.525285 | 1.567643 |
| ko00750//Vitamin B6 metabolism | 0.980975 | 1.361214 |
| ko00740//Riboflavin metabolism | 1.013079 | 1.287438 |
| ko00730//Thiamine metabolism | 1.144587 | 2.128749 |
| ko00710//Carbon fixation in photosynthetic organisms | 1.307151 | 2.031151 |
| ko00670//One carbon pool by folate | 2.501801 | 1.886382 |
| ko00660//C5-Branched dibasic acid metabolism | -3.64386 | 3.531693 |
| ko00650//Butanoate metabolism | 1.070827 | 3.284424 |
| ko00640//Propanoate metabolism | 1.692095 | 1.76362 |
| ko00630//Glyoxylate and dicarboxylate metabolism | 0.947906 | 1.705131 |
| ko00620//Pyruvate metabolism | 0.815258 | 1.873939 |
| ko00604//Glycosphingolipid biosynthesis - ganglio series | 0.962291 | 1.999611 |
| ko00603//Glycosphingolipid biosynthesis - globo and isoglobo series | 1.150622 | 0.568215 |
| ko00600//Sphingolipid metabolism | 0.605954 | 2.043934 |
| **Pathway Name** | **CK** | **IBA300** |
| ko00592//alpha-Linolenic acid metabolism | 1.650235 | 1.207351 |
| ko00591//Linoleic acid metabolism | 0.655914 | 1.752079 |
| ko00590//Arachidonic acid metabolism | 1.525008 | 1.98365 |
| ko00565//Ether lipid metabolism | 0.958827 | 1.278085 |
| ko00564//Glycerophospholipid metabolism | 1.265825 | 2.169772 |
| ko00563//Glycosylphosphatidylinositol (GPI)-anchor biosynthesis | 0.317769 | 1.589839 |
| ko00562//Inositol phosphate metabolism | 0.721291 | 1.218164 |
| ko00561//Glycerolipid metabolism | 0.876562 | 1.848519 |
| ko00531//Glycosaminoglycan degradation | 2.059861 | 1.559395 |
| ko00520//Amino sugar and nucleotide sugar metabolism | 1.401533 | 2.221641 |
| ko00515//Mannose type O-glycan biosynthesis | 0.526556 | 1.460623 |
| ko00514//Other types of O-glycan biosynthesis | 1.10534 | 1.5725 |
| ko00511//Other glycan degradation | 1.105116 | 1.116371 |
| ko00510//N-Glycan biosynthesis | 1.057319 | 1.497939 |
| ko00500//Starch and sucrose metabolism | 1.47532 | 1.729918 |
| ko00480//Glutathione metabolism | 1.254772 | 1.946483 |
| ko00460//Cyanoamino acid metabolism | 1.148971 | 1.435003 |
| ko00450//Selenocompound metabolism | 2.40108 | 2.175781 |
| ko00440//Phosphonate and phosphinate metabolism | -0.55503 | 2.048487 |
| ko00430//Taurine and hypotaurine metabolism | 1.116967 | 1.597858 |
| ko00410//beta-Alanine metabolism | 1.762436 | 1.697019 |
| ko00402//Benzoxazinoid biosynthesis | 1.484989 | 0.49553 |
| ko00400//Phenylalanine, tyrosine and tryptophan biosynthesis | 1.197491 | 0.946259 |
| ko00380//Tryptophan metabolism | 1.159663 | 1.641375 |
| ko00360//Phenylalanine metabolism | 1.017935 | 1.382203 |
| ko00350//Tyrosine metabolism | 1.237355 | 1.632062 |
| ko00340//Histidine metabolism | -0.96233 | 1.847576 |
| ko00330//Arginine and proline metabolism | 0.911101 | 1.673687 |
| ko00310//Lysine degradation | -0.38913 | 1.398948 |
| ko00300//Lysine biosynthesis | 1.777816 | 0.271134 |
| ko00290//Valine, leucine and isoleucine biosynthesis | 1.356543 | 0.767742 |
| ko00280//Valine, leucine and isoleucine degradation | 0.522988 | 1.22073 |
| ko00270//Cysteine and methionine metabolism | 1.317067 | 2.294268 |
| ko00261//Monobactam biosynthesis | 0.122444 | 1.790337 |
| ko00260//Glycine, serine and threonine metabolism | 0.950836 | 1.439793 |
| ko00250//Alanine, aspartate and glutamate metabolism | 1.13463 | 2.24793 |
| ko00240//Pyrimidine metabolism | 0.956483 | 1.583733 |
| ko00232//Caffeine metabolism | 5.236638 | 3.123383 |
| ko00230//Purine metabolism | 1.258125 | 1.746474 |
| ko00220//Arginine biosynthesis | 1.29094 | 1.059311 |
| ko00196//Photosynthesis - antenna proteins | 0.801312 | 2.101445 |
| **Pathway Name** | **CK** | **IBA300** |
| ko00195//Photosynthesis | 0.876629 | 2.197603 |
| ko00190//Oxidative phosphorylation | 1.325512 | 1.982602 |
| ko00130//Ubiquinone and other terpenoid-quinone biosynthesis | 2.123788 | 1.939391 |
| ko00100//Steroid biosynthesis | 1.952249 | 3.382544 |
| ko00073//Cutin, suberine and wax biosynthesis | 1.4004 | 2.138886 |
| ko00072//Synthesis and degradation of ketone bodies | 2.2435 | 2.168081 |
| ko00071//Fatty acid degradation | 1.600554 | 1.996225 |
| ko00062//Fatty acid elongation | 0.722015 | 1.979645 |
| ko00061//Fatty acid biosynthesis | 1.455039 | 1.346513 |
| ko00053//Ascorbate and aldarate metabolism | 1.58019 | 1.831276 |
| ko00052//Galactose metabolism | 1.048041 | 1.949978 |
| ko00051//Fructose and mannose metabolism | 0.836303 | 1.902684 |
| ko00040//Pentose and glucuronate interconversions | 1.236698 | 1.501583 |
| ko00030//Pentose phosphate pathway | 0.985901 | 1.890445 |
| ko00020//Citrate cycle (TCA cycle) | 0.725204 | 1.716275 |
| ko00621//Pyruvate metabolism | 1.692408 | 1.947744 |

**Table S5.** *ArARF* genes encoding ARF proteins along with their molecular details.

| **Gene** | **Gene ID** | **Length(aa)** | **pI** | **MW(kDa)** | **Domain** |
| --- | --- | --- | --- | --- | --- |
| *ArARF1* | CL12499.Contig1_All | 682 | 6.08 | 75.74 | DBD,ARF,CTD |
| *ArARF2* | CL1603.Contig1_All | 848 | 6.34 | 94.59 | DBD,ARF,CTD |
| *ArARF2* | CL999.Contig2_All | 743 | 6.37 | 83.4 | DBD,ARF,CTD |
| *ArARF3* | CL8458.Contig2_All | 754 | 6.12 | 81.74 | DBD,ARF |
| *ArARF4* | CL4652.Contig2_All | 796 | 6.31 | 88.37 | DBD,ARF |
| *ArARF5* | CL11296.Contig1_All | 940 | 5.3 | 103.97 | DBD,ARF,CTD |
| *ArARF6* | CL6338.Contig1_All | 910 | 5.92 | 100.71 | DBD,ARF,CTD |
| *ArARF7* | Unigene23399_All | 914 | 6.23 | 101.6 | DBD,ARF,CTD |
| *ArARF8* | Unigene2668_All | 840 | 5.87 | 93.89 | DBD,ARF,CTD |
| *ArARF9* | CL12892.Contig1_All | 690 | 6.26 | 77.21 | DBD,ARF,CTD |
| *ArARF10* | CL3897.Contig1_All | 733 | 8.39 | 80.61 | DBD,ARF |
| *ArARF16* | CL7048.Contig1_All | 704 | 6.76 | 77.76 | DBD,ARF |
| *ArARF17* | CL5608.Contig3_All | 707 | 5.96 | 78.05 | DBD,ARF,CTD |
| *ArARF18-1* | Unigene5190_All | 612 | 5.95 | 67.17 | DBD,ARF |
| *ArARF18-2* | Unigene426_All | 612 | 5.95 | 67.17 | DBD,ARF |
| *ArARF19-1* | CL1642.Contig1_All | 1108 | 6.24 | 122.56 | DBD,ARF,CTD |
| *ArARF19-2* | CL1642.Contig5_All | 1108 | 6.24 | 122.52 | DBD,ARF,CTD |
| *ArARF19-3* | CL4170.Contig3_All | 1170 | ·6.19 | 131.19 | DBD,ARF,CTD |

## Supplementary Figures

**
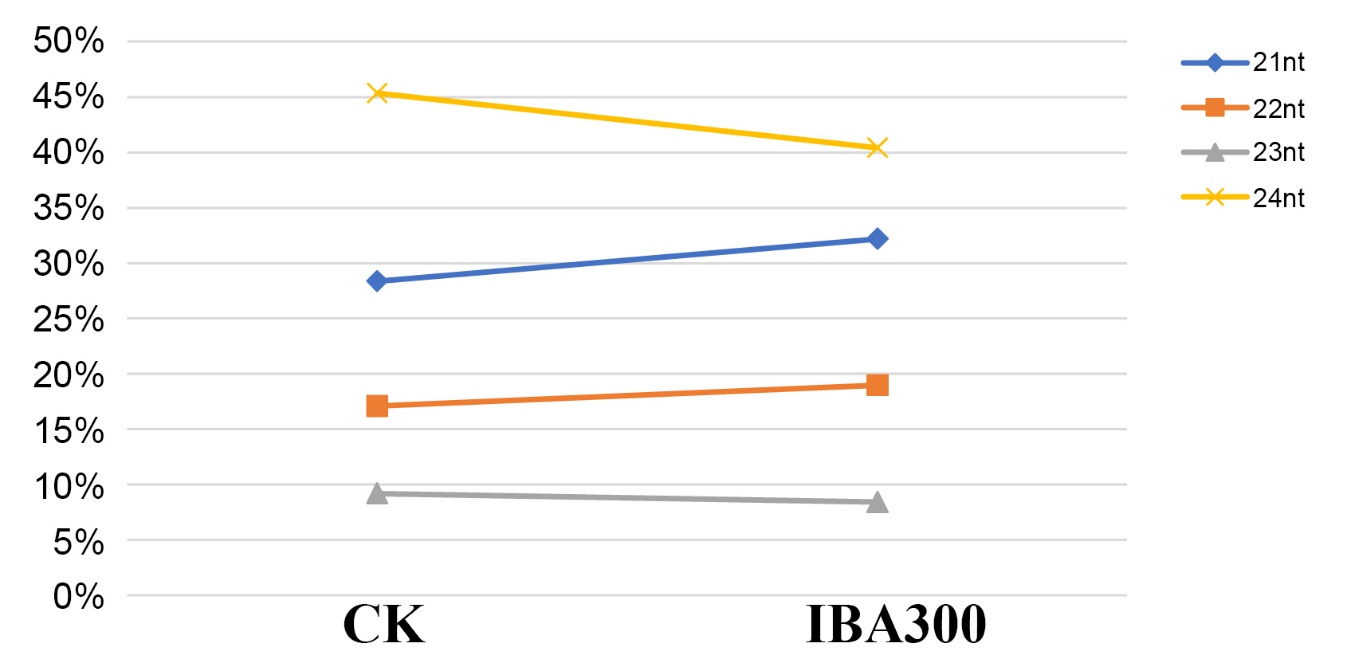
**

**Fig.S1. The distribution of 21–24 nt small RNAs (sRNAs) across the two sRNA libraries.**

**
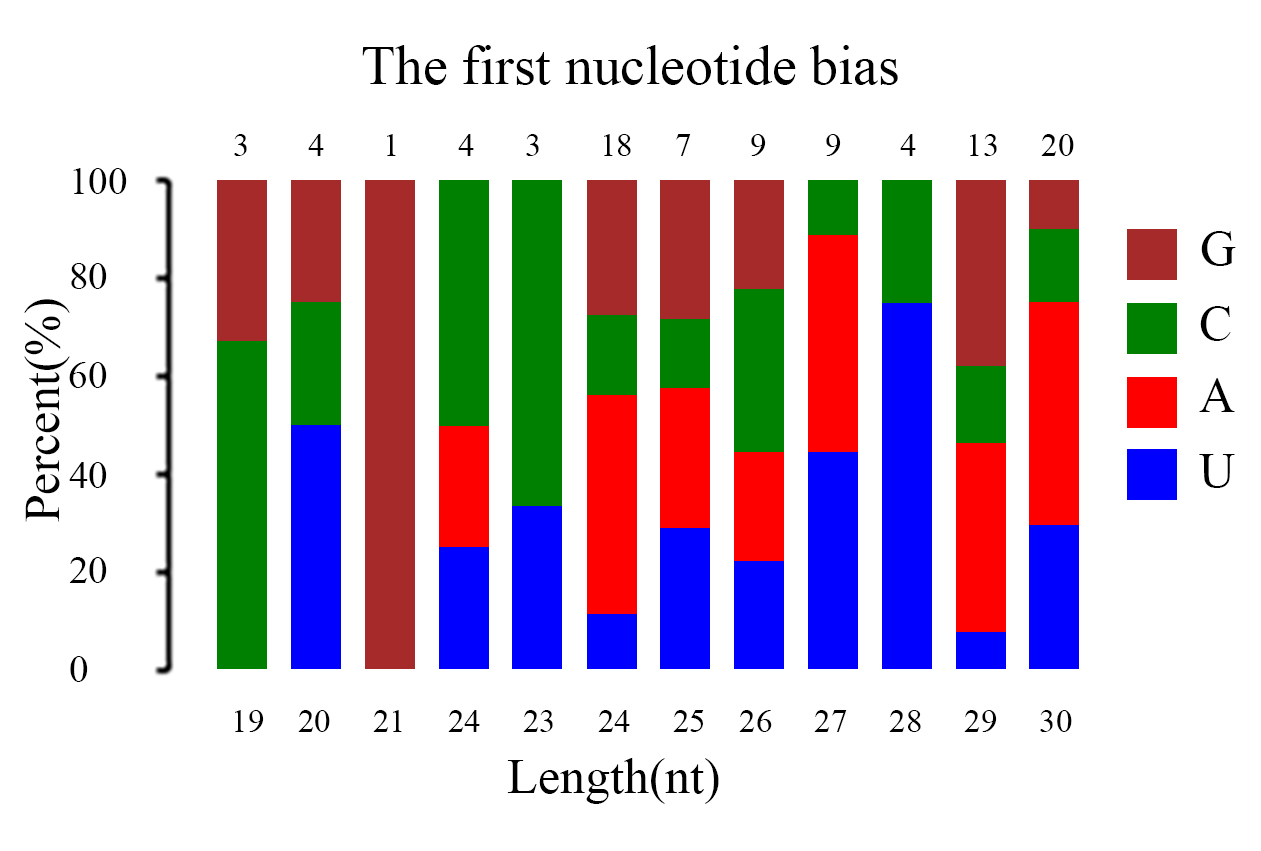
**

**Fig.S2. First nucleotide bias across the novel microRNAs.** X-axis represented length of miRNA (19–30 nt long), Y-axis showed the percent of the first nucleotide in each length miRNA.

**
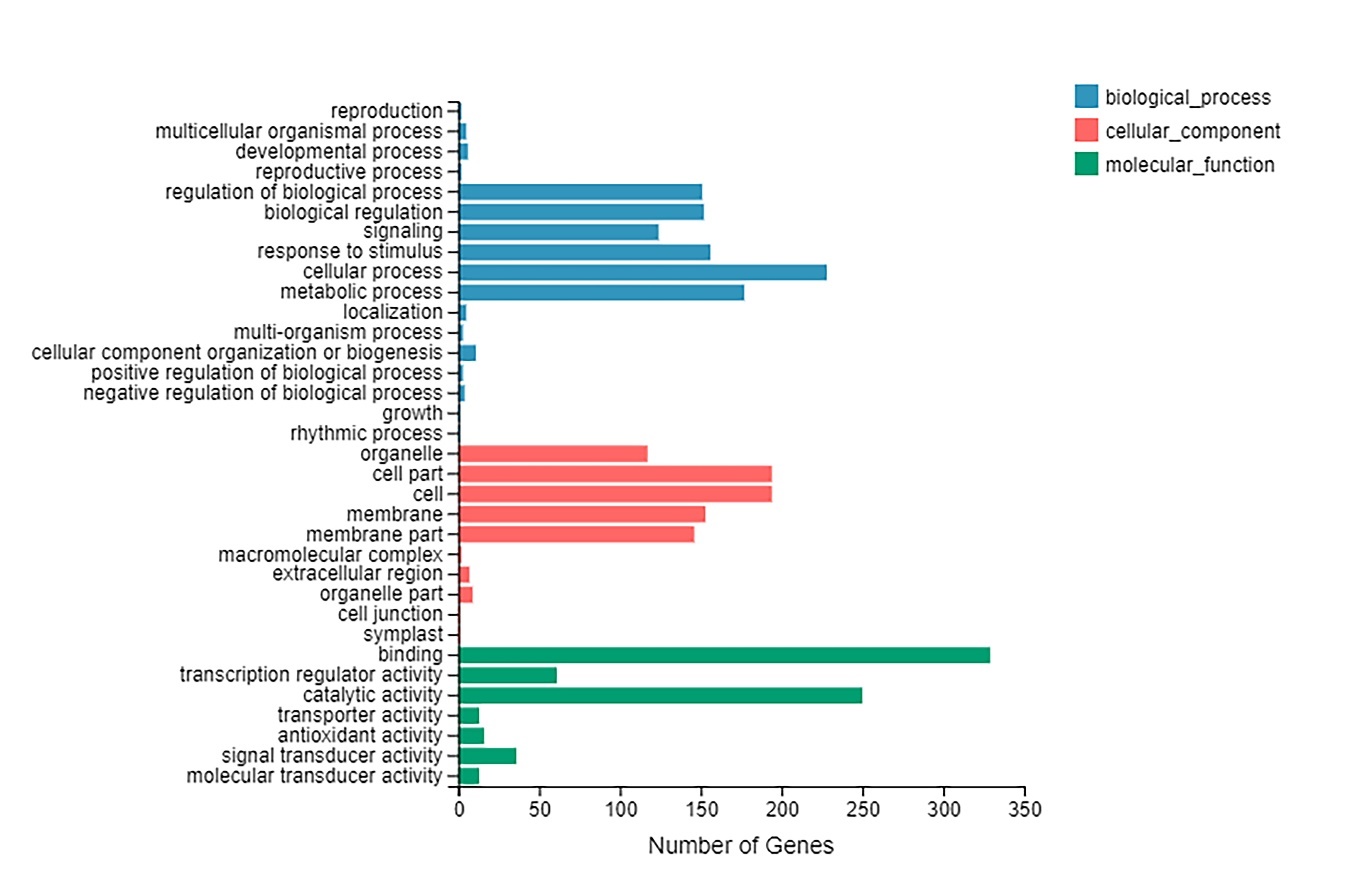
Fig.S3. GO annotation of the DEGs.**

**
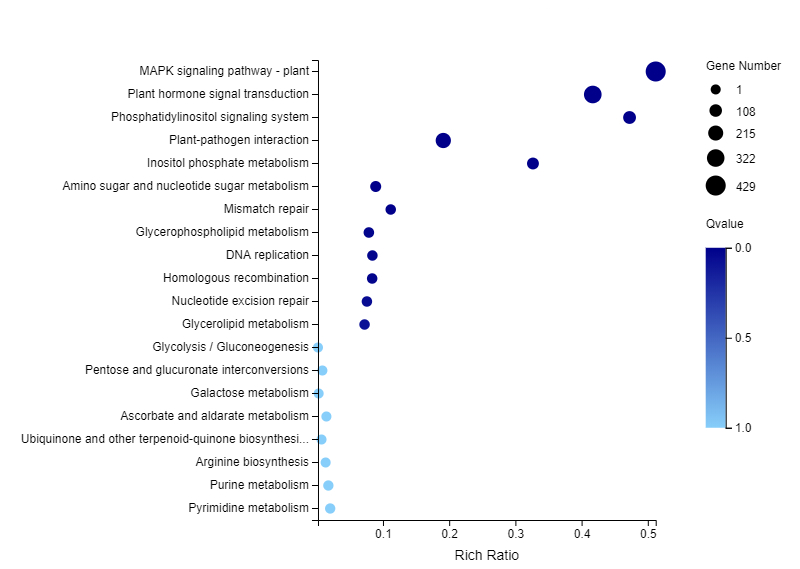
Fig.S4. KEGG pathway enrichment of the DEGs.**

**
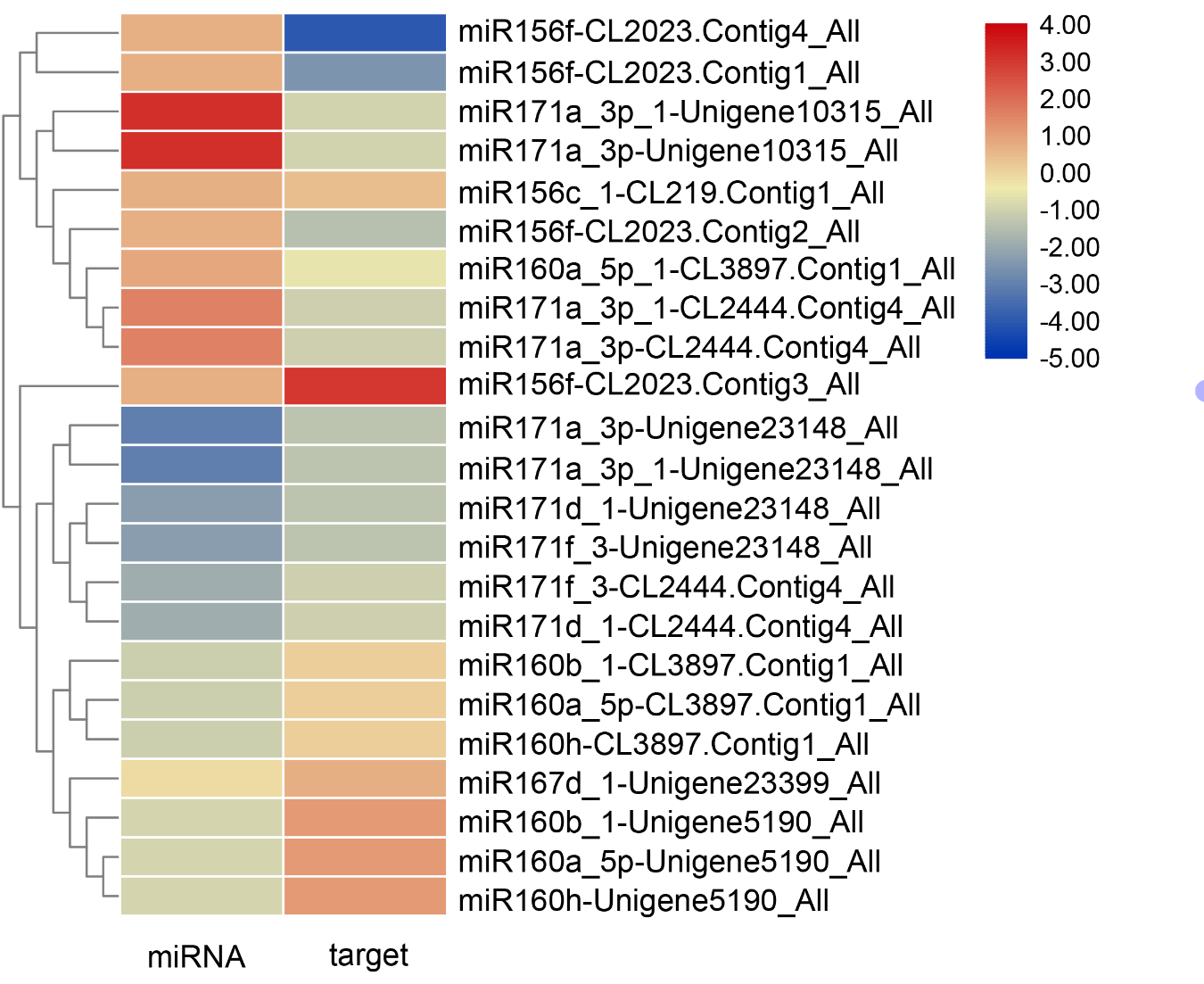
Fig.S5. Heat map showing the differentially expressed miRNAs and the corresponding 10 targets.** Upregulated genes/miRNAs were shown in red; downregulated genes/miRNAs were shown in blue.

**
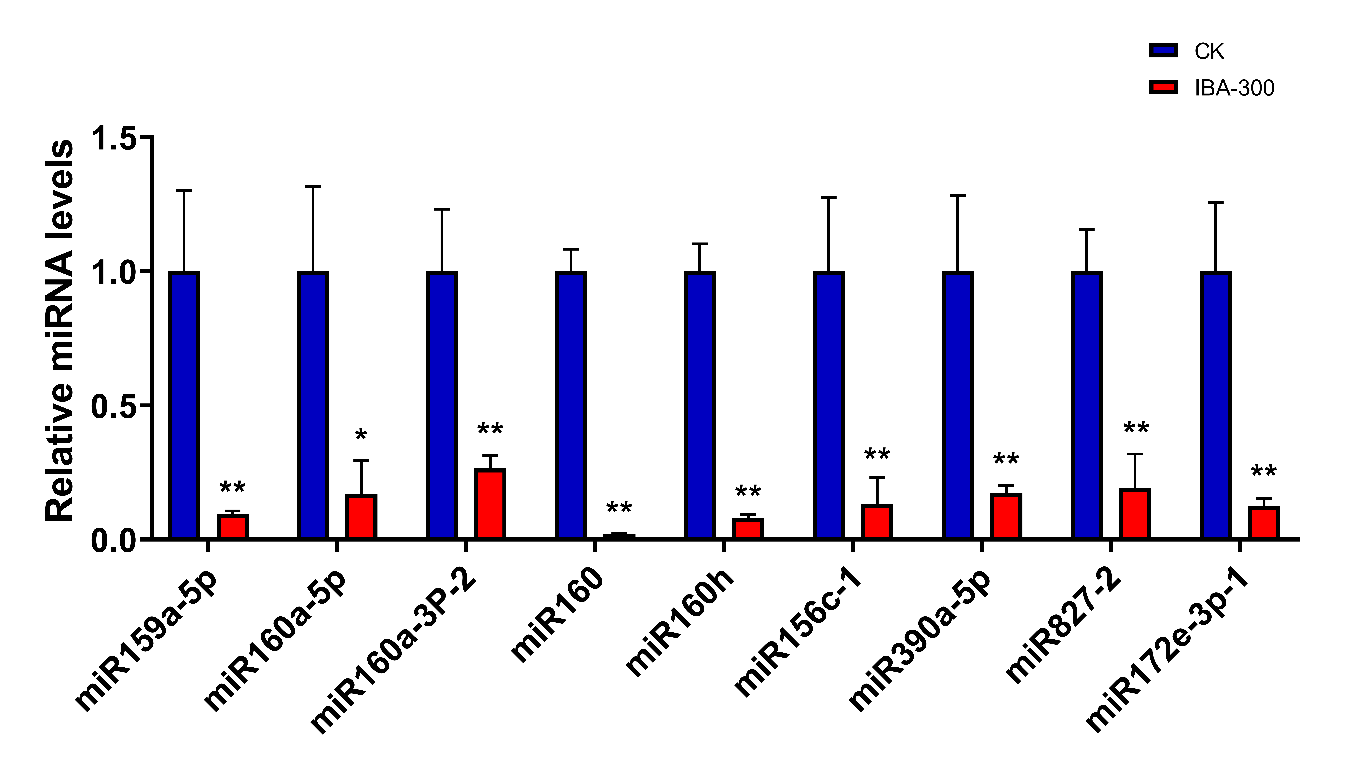
Fig.S6. Expression patterns of nine selected miRNAs in the adventitious roots of *A. rubrum* cuttings treated with auxin (IBA300) and untreated water (CK).** U6 snRNA was used as a reference for qRT-PCR, and miRNA expression in the control was set to 1.0. Mean values of three replicates were shown with standard error bars. “*” indicated significant difference at p-value < 0.05, “**” indicated significant difference at p-value < 0.01.

**
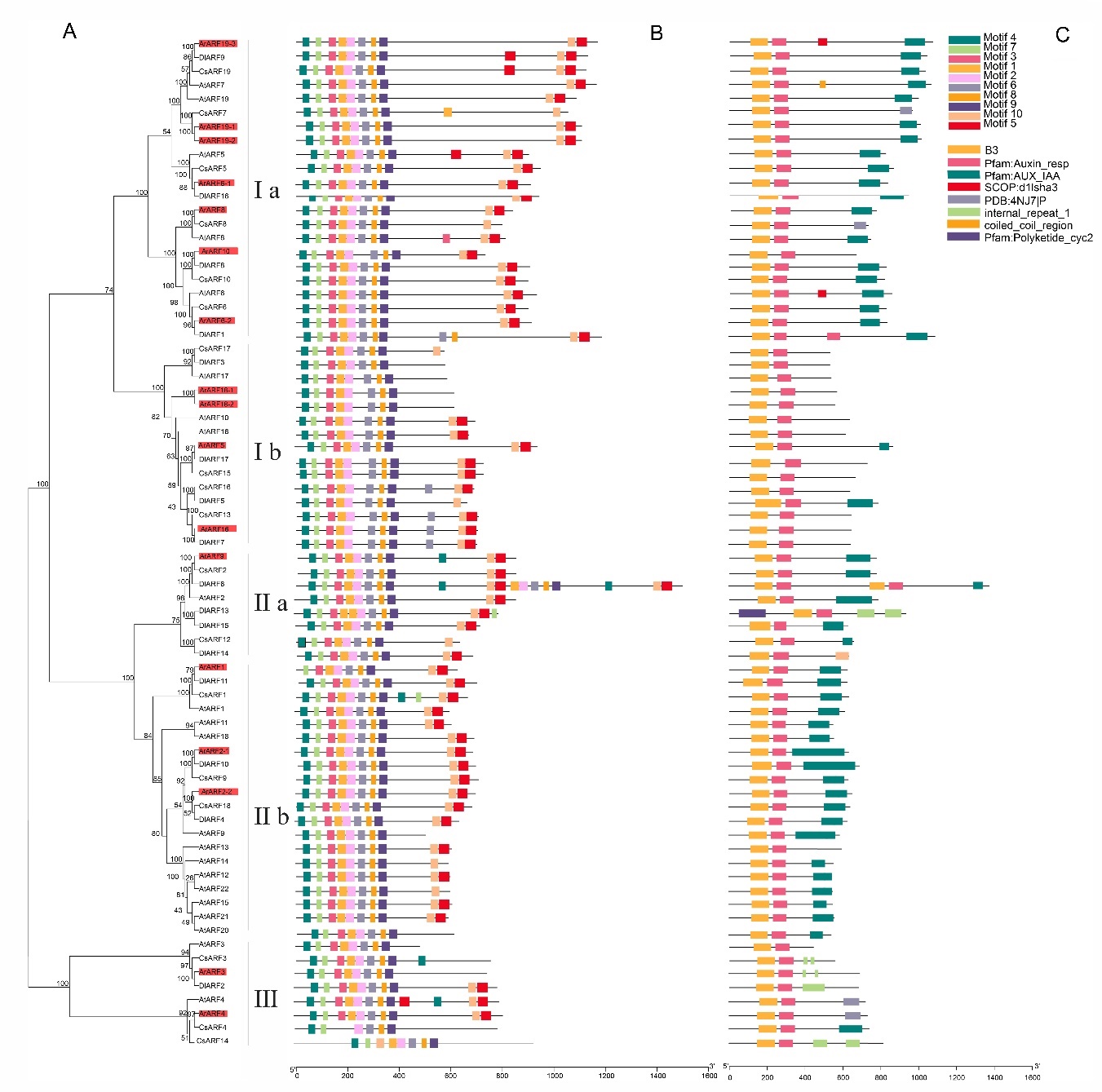
Figure S7. Neighboring-joining (NJ) phylogenies and representative conserved motif patterns in ARF proteins from *A. rubrum*,** **longan,** **citrus, and *A.thaliana*.** (A) A phylogenetic tree was constructed for full-length ARF proteins from four plant species, including *A.rubrum* (Ar), *Dimocarpus longan* (Di), citrus (Cs), *Arabidopsis thaliana* (At). (B) Distribution of ARF proteins of 10 motifs in four species. (C) Conservative domain of ARF proteins of 10 motifs in four species.

**
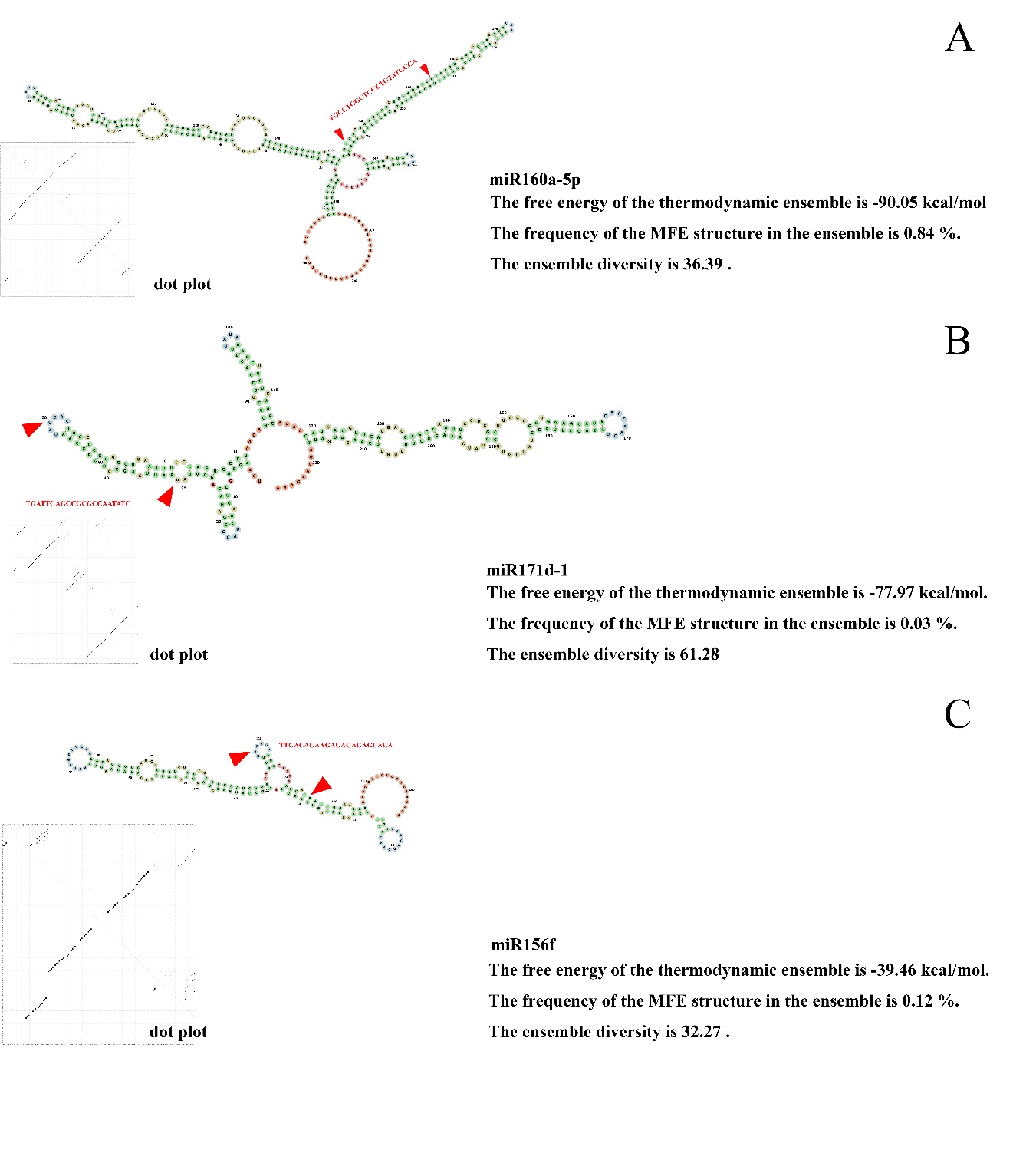
Fig. S8. The precursor sequences of three miRNAs.**

**
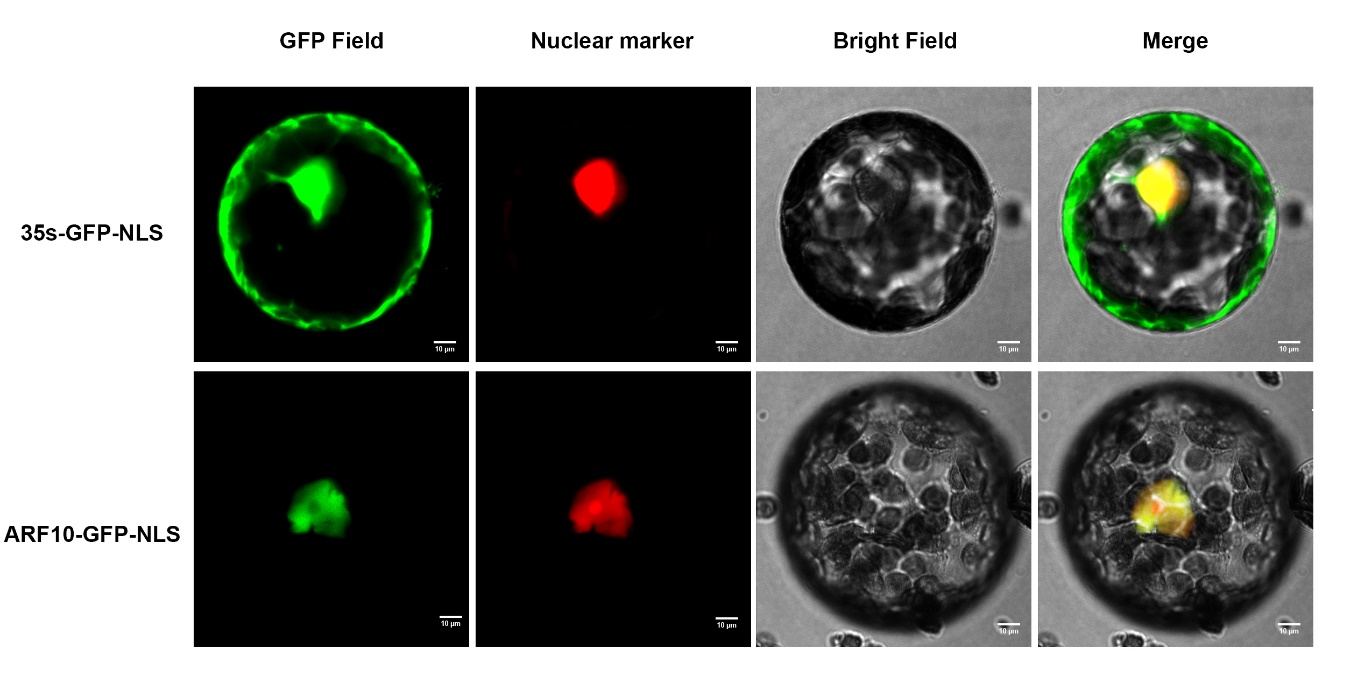
**

**Fig. S9. Subcellular localization of ArARF10.**

**
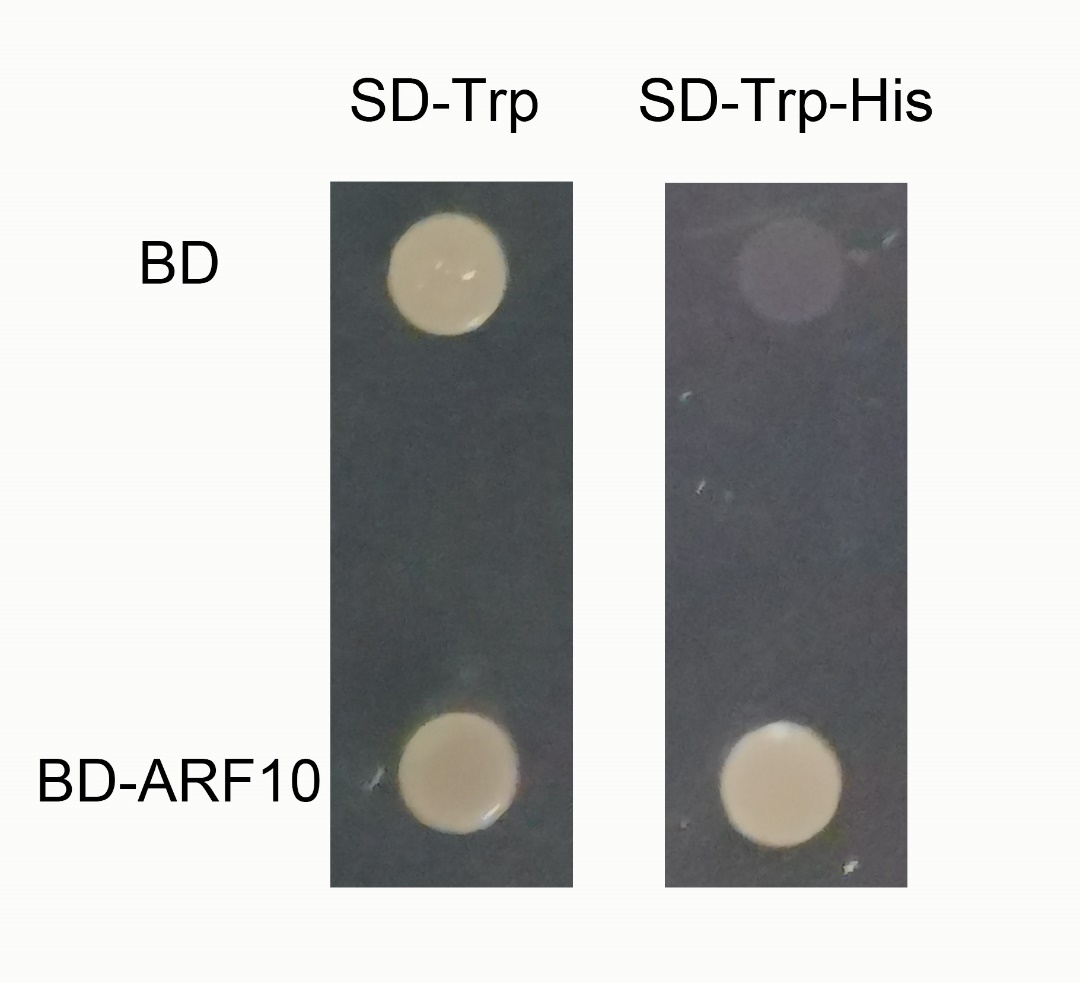
**The merged pictures of the green fluorescence channel, nuclear marker and the corresponding bright field are shown. Cells expressing the GFP was used as a control. Scale bar, 10μm.

**Fig. S10. Transcriptional activation** **analysis of ArARF10.**

**
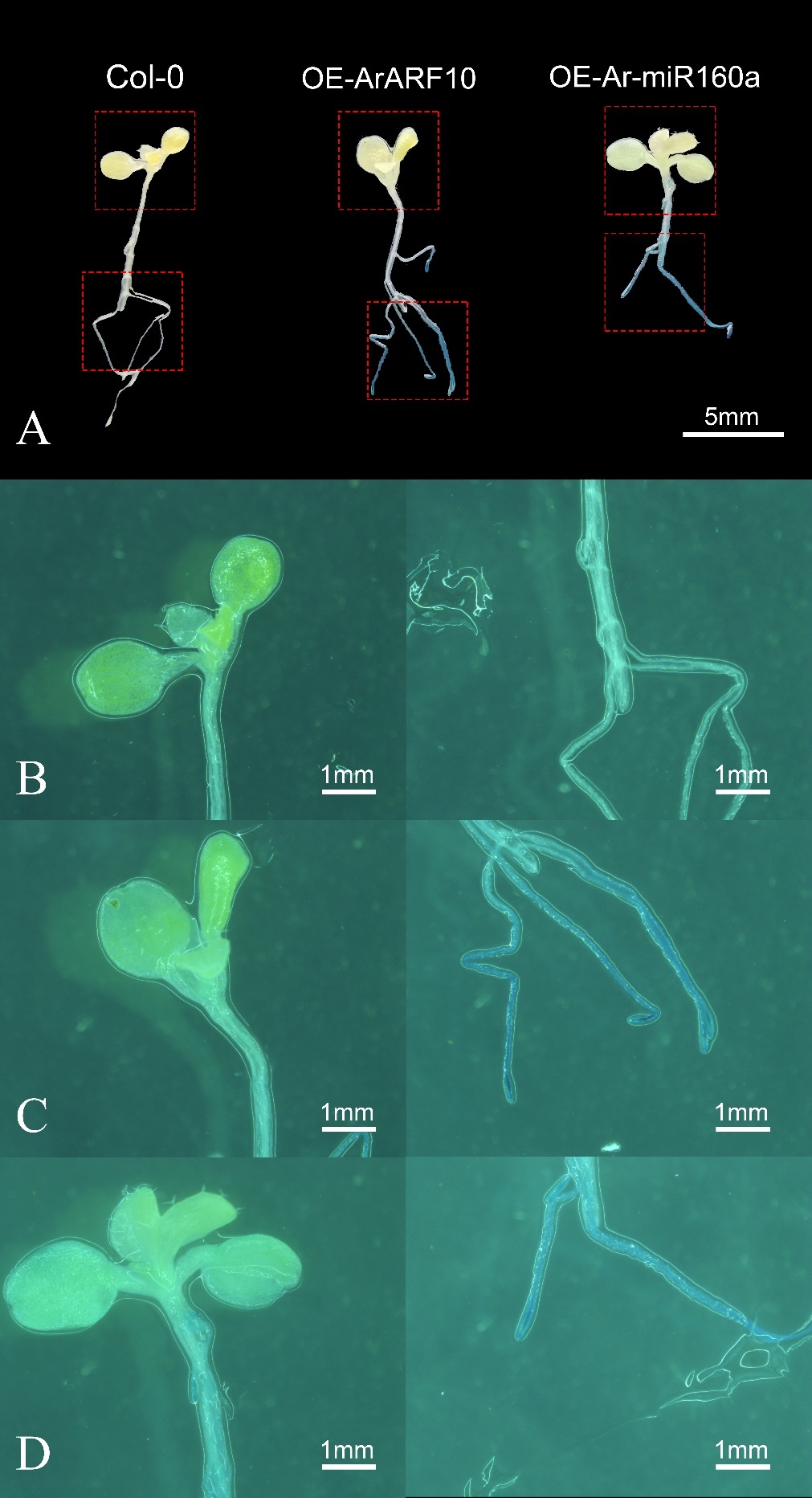
**

**Fig.S11. GUS-stained seedlings, showing successful expression of the *Ar-miR160* and its target gene (*ArARF10*).** (A) Hypocotyls and ARs of OE-*ArARF10* and OE-*Ar-miR160a* plants showed blue color compared with Col-0 plants, and the inserted GUS gene expression indicated successful gene transformation. Scale bar,5 mm (B) Col-0 plants showed no change in color after GUS staining. (C) OE-*ARARF10* plants turned blue after staining and changed significantly in the AR site. (D) OE-*AR-miR160a* plants turned blue and the color change of AR was obvious, Scale bar,1mm.
